# Identification via Virtual Screening of Emissive Molecules with a Small Exciton–Vibration Coupling for High Color Purity and Potential Large Exciton Delocalization

**DOI:** 10.1021/acs.jpclett.3c00749

**Published:** 2023-04-27

**Authors:** Xiaoyu Xie, Alessandro Troisi

**Affiliations:** Department of Chemistry, University of Liverpool Liverpool L69 3BX, U.K.

## Abstract

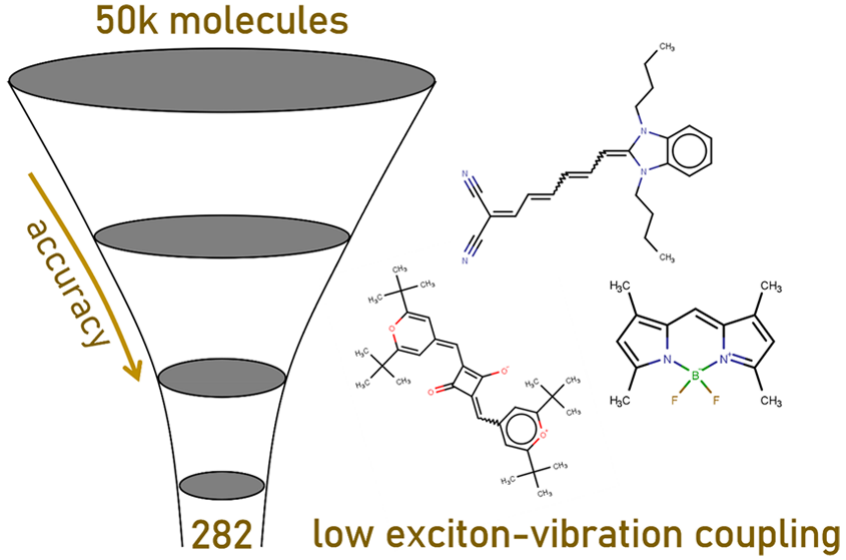

A sequence of quantum chemical computations of increasing accuracy was used in this work to identify molecules with small exciton reorganization energy (exciton–vibration coupling), of interest for light emitting devices and coherent exciton transport, starting from a set of ∼4500 known molecules. We validated an approximate computational approach based on single-point calculations of the force in the excited state, which was shown to be very efficient in identifying the most promising candidates. We showed that a simple descriptor based on the bond order could be used to find molecules with potentially small exciton reorganization energies without performing excited state calculations. A small set of chemically diverse molecules with a small exciton reorganization energy was analyzed in greater detail to identify common features leading to this property. Many such molecules display an A–B–A structure where the bonding/antibonding patterns in the fragments A are similar in HOMO and LUMO. Another group of molecules with small reorganization energy displays instead HOMO and LUMO with a strong nonbonding character.

The different equilibrium geometries of the ground and excited state of a molecule cause the emergence of vibronic progression in absorption and emission electronic spectra. Such deformation of the excited state due to the vibronic coupling and quantified by the exciton reorganization energy is critically important for several practical applications. In luminescent organic semiconductor materials used, for example, to develop organic light-emitting diodes (OLEDs),^1−3^ it is highly desirable to have light emission with a relatively narrow bandwidth, i.e., a high color purity. From the microscopic point of view, this means that the emissive molecules should have the smallest possible vibronic coupling to suppress vibronic sidebands that reduce color purity.^4,5^ For different applications in photovoltaics^6−8^ but also quantum technology,^9^ it is desirable to promote long-range exciton transport in molecular materials. In different works,^10,11^ it was observed that the typical value of the exciton-vibration coupling found in medium-sized molecules is sufficient to localize the exciton on a single molecule and reduce the exciton diffusion. However, there are known cases like dicyanovinyl-capped *S*,*N*-heteropentacene (DCVSN5),^10,12^ where the vibronic coupling terms are small enough for the system to sustain very delocalized excitons.^11,13−15^ High color purity emitters and molecular materials with delocalized excitons are further related because a strong oscillator strength for the lowest excited state is required for both applications.

The identification of novel molecules with a small vibronic coupling is challenging because there are no practical design rules. A recent exploration of this issue was carried out by Penfold et al.^5^ The authors investigated the emission full-width at half-maximum (fwhm) using the displaced harmonic oscillator model (DHO) for 27 typical molecules including truxene and a sample of multiresonance, charge transfer, and polycyclic aromatic molecules. Based on the DHO model, they provide a strategy for rapid prediction of the color purity of luminescent organic molecules. Besides this, they found a strong correlation between fwhm and the nuclear gradient of the excited state potential.

To the best of our knowledge, there have not been many studies aiming at the identification of low reorganization energy compounds via high-throughput virtual screening (HTVS). When this approach is used to explore a large set of compounds that have not been designed to have certain characteristics, one can discover very different molecules with the desirable electronic properties (e.g., for singlet fission,^16^ temperature-activated delayed fluorescence,^17^ electron acceptors for photovoltaic applications^18,19^). A key advantage of a diverse set of molecules with a given uncommon property is that one can identify structure–property relationships and, therefore, a novel approach to designing new molecules. The objective of this work is to construct a large set of molecules with small excitonic reorganization energy through a sequence of increasingly more accurate virtual screening steps. We then analyze the results drawing general conclusions on the features that impart low excitonic reorganization energy to organic molecules.

Conventionally, the calculation of the reorganization energy needs the equilibrium structure of initial and final electronic states (e.g., S_1_ and S_0_ states for the emission processes, as shown in Figure 1). The most common way to evaluate reorganization energy (a measure of exciton-vibration coupling) is by using its definition, i.e., the four-point approach as shown below,
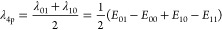
1Here, *E*_*ij*_ is the energy of state *i* at the optimized geometry of state *j*, illustrated in Figure 1. This approach requires the geometry optimization of S_0_ and S_1_. The latter, in particular, is computationally very expensive and unsuitable for HTVS.

**Figure 1:**
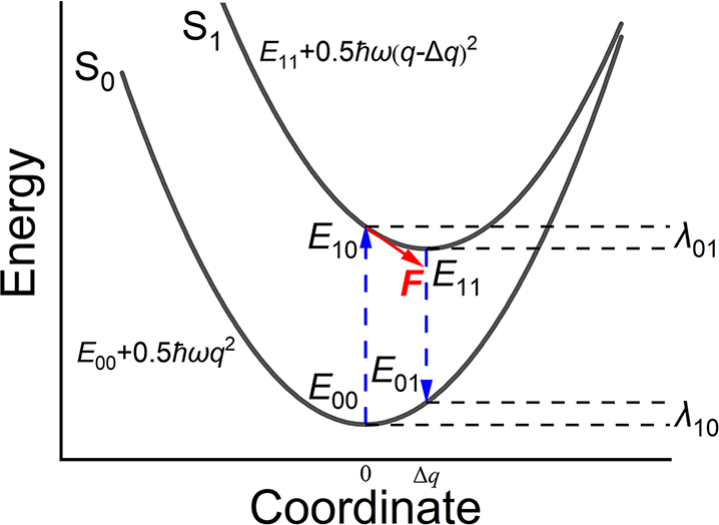
Illustration for calculating reorganization energy (λ) using 1D potential energy surfaces. Under the harmonic and Condon approximation,  and *F* = ℏωΔ*q*. Equations in the manuscript are for potential energy surfaces of higher dimensions.

A convenient approximation, often invoked when many calculations of the reorganization energy are needed^20^ and also used to study color purity^5^ can be used to evaluate reorganization energy for the S_0_ →S_1_ transition.^21,22^ Under the Condon and the harmonic approximations, the computation of S_1_ state force at S_0_ geometry instead of S_1_ optimization is performed to calculate the dimensionless displacement Δ*q*_*i*_ of a normal mode *i*,
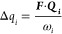
2where *F* is the mass-weighted force vector of the S_1_ state at S_0_ geometry, *Q*_***i***_ and ω_*i*_ is the normalized displacement and frequency of the mode *i*, respectively. The total reorganization energy can be calculated as
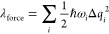
3

For this approach, geometry optimization of S_0_, frequency analysis of S_0_, and force calculation of S_1_ state are needed. Therefore, geometry optimization of the S_1_ state, the most expensive process of the four-point method, can be avoided. Based on preliminary calculations involving 400 molecules at the B3LYP/6-31g(d) level (see Supporting Information for the details of our preliminary data set), about 80% of the computational cost can be saved via eq 3 compared to eq 1.

The accuracy of the approximation of eq 3 was evaluated, comparing λ_force_ and λ_4p_ for our preliminary data set. As shown in Figure 2, the force approach underestimates the evaluation of λ, especially for large λ due to the breaking of the harmonic and Condon approximation. However, it shows good agreement with λ_4p_ for small λ and, therefore, it is ideal as a first step of screening. For example, the criterion λ_force_ < 0.25 eV will identify 98% of the molecules with λ_4p_ < 0.25 eV, and only 25% of the molecules with λ_force_ < 0.25 eV have λ_4p_ > 0.25 eV.

**Figure 2:**
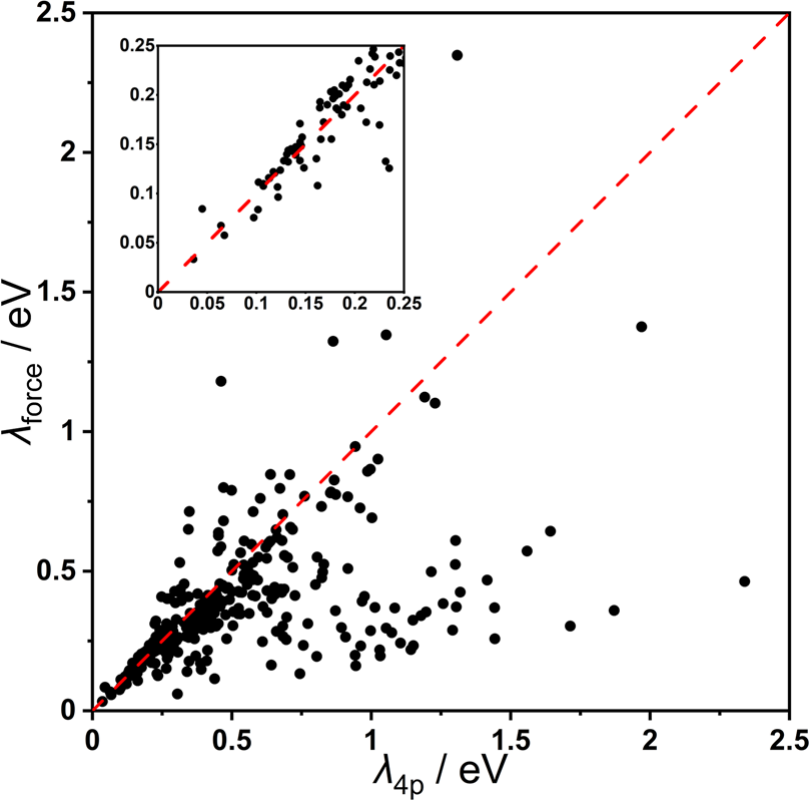
Comparison of total reorganization energy between the force approach (λ_force_) to the common four-point approach (λ_4*p*_). The subfigure shows zoom-in results of small λ (the Pearson’s *R* = 0.88 for this subset).

We considered 4476 molecules extracted from the data set of computed excited states of molecules reported in ref (23), which includes 48182 molecules with experimentally known crystal structures from the Cambridge Structural Database.^24^ The smaller set was obtained by imposing *E*_*S*_2__ – *E*_*S*_1__ > 0.05 eV (to reduce the chance that S_1_ and S_2_ are interchanged going from theory to experiment), *E*_*S*_1__ < 3.65 eV (to remove less technologically interesting chromophores), removing molecules with more than 100 atoms (to improve throughput), and considering only molecules with S_1_ oscillator strength larger than 0.5 (since a bright emissive state is required by all the applications mentioned in the introduction, and the finding can be tested more easily). It should be noted that this work only directly addresses molecular rather than solid state properties, as it was noted that the extent of exciton delocalization is influenced not only by the excitonic coupling but also the nonlocal exciton–phonon coupling.^25,26^

The screening is performed in three layers, with each subsequent layer involving a subset of molecules from the previous layer and higher accuracy:

(i) For the 4476 molecules, the force approach (eq 3) was applied to evaluate λ_force_. The (TDDFT) M06-2*X*/3-21g* calculation level was applied for these calculations using the G16 package.^27^ A reduced convergence criterion was applied for S_0_ geometry optimization to further speed up the calculations without accuracy loss (these approximations are validated in the Supporting Information). For frequency analysis and the normal modes used in eq 2, we ignored the modes with low imaginary frequency since some systems cannot be optimized to the exact global minimum.
(ii) Molecules from the screening in section i that have λ_force_ < 100.0 meV were selected for the four-point approach calculation (eq 1) using the same level of theory.
(iii) Molecules with a small reorganization energy calculated in section ii (λ_4p_ < 125.0 meV) were grouped into distinct sets based on the similarity of molecular structures. For each set, one or two molecules were selected as representatives, and the calculation of λ_4p_ was repeated at the higher level, M06-2X/def2-SVP.

The list of molecules considered with their optimized geometries and the key parameters discussed in this work are given in a public repository.^28^

The distribution of the computed λ_force_ is reported in Figure 3a. The median value is 287.9 meV; a reasonable subset of molecules to be investigated more accurately are those with λ_force_ < 100 meV considering that molecules typically associated with small reorganization energy (e.g., Y6, DCVSN5, and PDI) display values in the range of 125–180 meV^11^ (it is worth mentioning that papers discussing exciton transport^10,11,29^ define the exciton reorganization energy for the transfer of exciton between two molecules–which is double the value as defined in this work). There are 358 molecules with λ_force_ < 100 meV for which the four-point method is applied to get more accurate reorganization energy. The results are compared to the force approach in Figure 3b, and similar to our preliminary test in Figure 2, λ_force_ is a lower bound for λ_4p_ and can be used to identify potential candidates with small λ_4p_.

**Figure 3:**
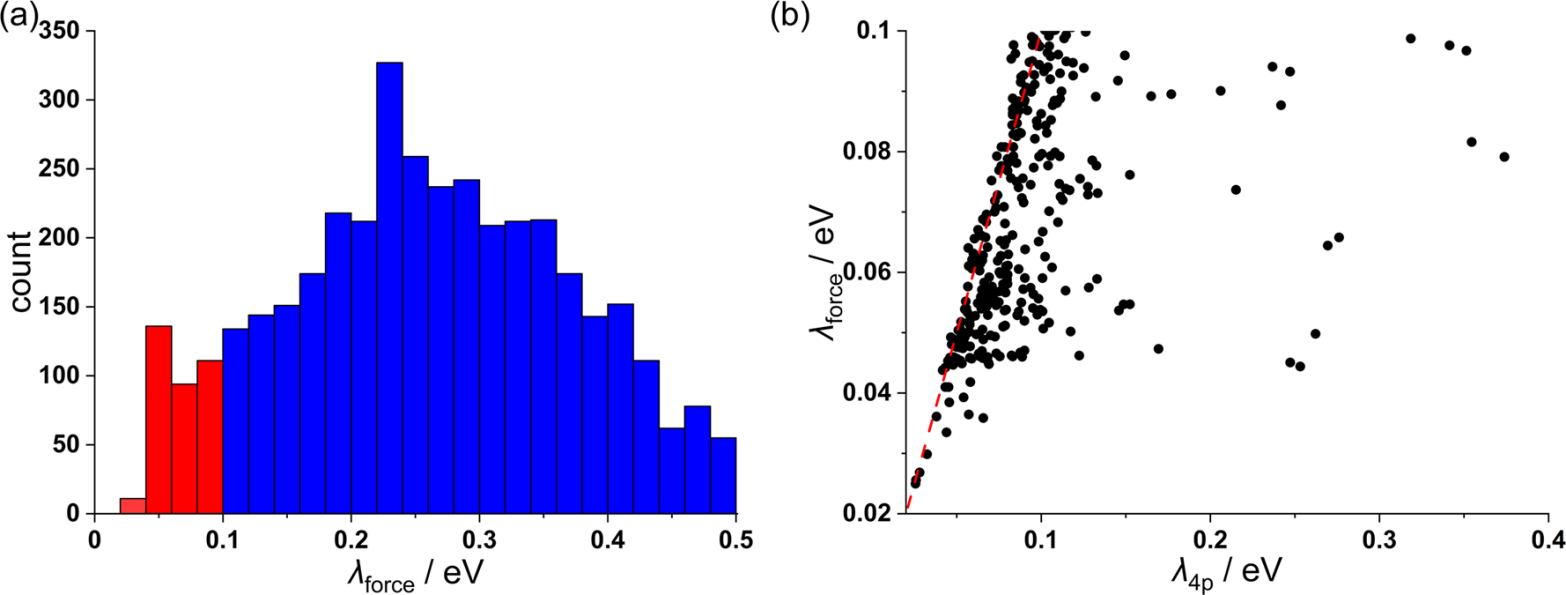
(a) Distribution of reorganization energy λ_force_ in the energy region [0, 0.5] eV (**layer i** calculation) with red bars presenting the systems with small λ_force_ for **layer ii**. (b) Comparison of reorganization energies between eq 1 (λ_4p_) and eq 3 (λ_force_). The red dashed line indicates the λ_force_ = λ_4p_ condition.

Before discussing specific cases, it is useful to report more general correlations in the data set. It is expected that frontier delocalized orbitals are associated with smaller reorganization energy, and this is true, for example, for the polyacene series.^10^ However, there is only a very weak correlation between measures of delocalization of frontier orbitals (e.g., inverse participation ratio) and reorganization energy (correlation coefficient *R* = 0.19), as reported in Supporting Information, highlighting the importance of using large data set to determine structure–property relations that are truly useful in practice. A simple orbital-based parameter with a stronger correlation with the reorganization energy is the (squared) total bond order difference (BOD) between S_0_ and S_1_ by assuming that S_1_ is HOMO → LUMO excitation.
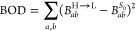
4where *B*_*ab*_^*S*_0_^ is the bond order between atoms *a* and *b* of in the ground state and *B*_*ab*_^H→L^ is the same quantity for the singlet HOMO → LUMO excitation configuration. The definitions of these parameters are taken from ref.^30−32^ and are reproduced in the Supporting Information. The summation extends over all pairs of chemically connected atoms. This idea, related to the bonding/antibonding pattern of HOMO/LUMO, is not novel and has already been covered in the literature.^11,33−35^ For example, the bond-order–bond-length (BOBL) relationship was used to elucidate reorganization energy in recent work.^35^ However, the predictive ability of this descriptor based on a large data set has not been presented before and, as we have seen, this is critical to establish its usefulness.

In our data set, 74% of the S_1_ excited states are dominated (>90% weight) by the HOMO → LUMO excitation. In such a case, the BOD parameter should be a more accurate predictor of the reorganization energy, as illustrated by Figure 4a, where the data points of BOD and λ_force_ are labeled according to the HOMO–LUMO weight in S_1_. While BOD cannot be used to predict accurately the λ_force_ in general, the value of BOD sets a lower bound to the value of the corresponding λ_force_. For example, BOD should be smaller than 0.2 if one is searching for molecules with λ_force_ < 100 meV. The predictivity is low since **2270 in 4476** molecules have BOD < 0.2, while only **358 molecules** have λ_force_ < 100 meV, but it should be noted that evaluating BOD does not require the calculation of the excited state. If the excited state composition is known (many databases now collect vertical excited states properties of molecules),^23,36,37^ the BOD parameters can be used for molecules dominated by the HOMO–LUMO transition in the lowest excited states and the predictivity is substantially enhanced. For example, as shown in Figure 4b, when BOD is in the vicinity of 0.1, the predicted median λ_force_ is 160 meV and 50% of the computed reorganization energies are in the range between 120 and 185 meV.

**Figure 4:**
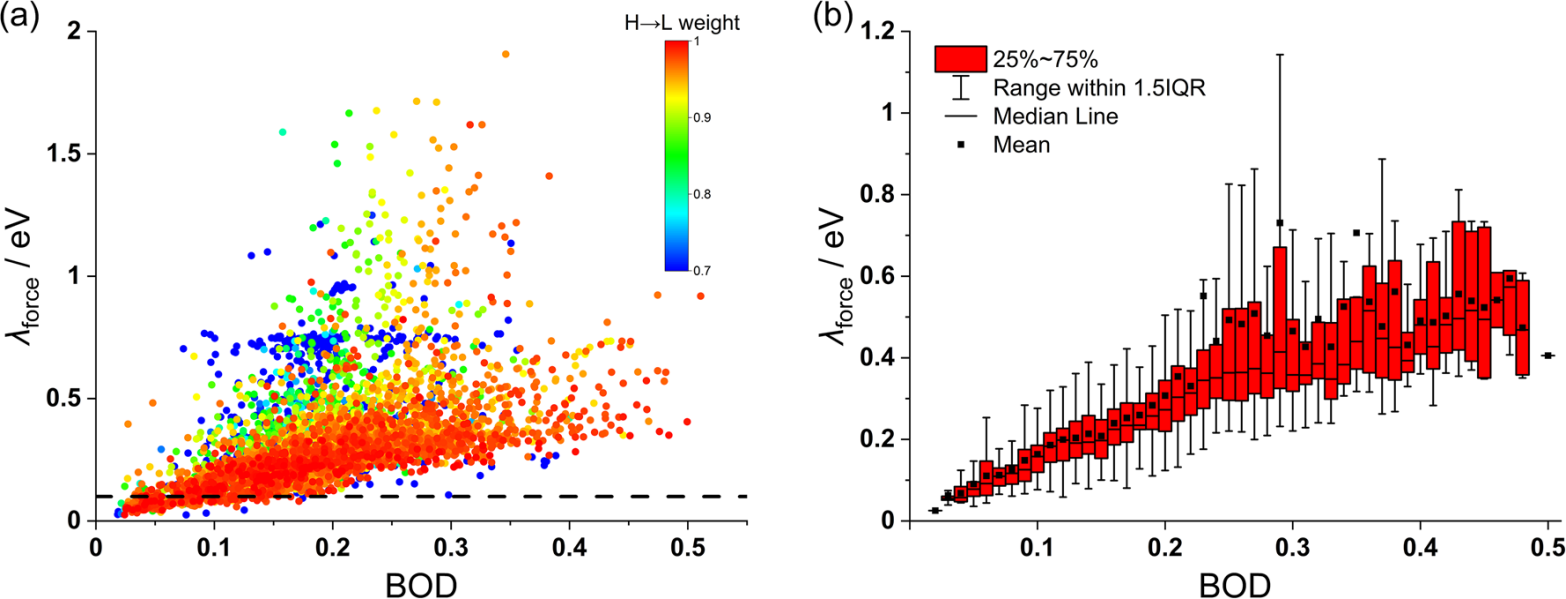
Relationship between BOD and reorganization energy for results in **layer i**. (a) All 4476 molecules (dashed line: λ_force_ = 0.1 eV) with data point color labeled according to the HOMO–LUMO excitation weight. (b) Box plot that shows the range of expected λ_force_ for intervals of BOD for molecules with HOMO–LUMO excitation weight larger than 0.9.

To give further insight into the results, we have looked individually at the 282 molecules in **layer ii** with small reorganization energy (λ_4p_ < 125.0 meV). We have noticed that many of such molecules share similar chemical features. We found it convenient to identify a representative of each class for a discussion of the origin of their small reorganization energy, as commonly done in this type of study.^16^ A very large number of molecules (196 molecules) belong to the class of **BODIPY** (boron dipyrromethenes) with the addition of the fluorine-substituted variant (19 molecules) or a variant with the substitution of nitrogen (2-ketopyrrole and β-alkyl-substituted dipyrrolyldiketone, 22 molecules). Using CSD identifiers, these three classes are represented by WEPGUU04, CIVNON, and IGUTIO, respectively, in Table 1 (chemical representation and frontier orbitals of the most interesting cases are given further below). BODIPY and its derivatives are clearly very well-known narrow emitters with many in-depth studies of their electronic characteristic.^38,39^ It is in many ways desirable as a verification of the screening approach to find molecules that are already known to have the sought characteristics. Similarly, 8 squaraine dye molecules are represented by VIFISEI in the table, as well as one similar structure known as croconaine dye (QALLOH). These systems were also well-studied and have sharp absorption and strong fluorescence emission at the near-infrared region, associated with a small reorganization energy.^40−44^ Furthermore, another group of 16 related molecules contain polyenes terminated by two −CN groups and is represented by CITGUG and CITCOA in the table. The remaining entries (OPOPOZ, PHTHCY01, QIQSER, WOJWIE, MUKKAE, PUTCEM, etc.) have 2–3 analogues in the search or appear to be isolated. The calculation of λ_4p_ is repeated at the higher level M06-2X/def2-SVP for 17 representative molecules, 13 of which remain of low reorganization energy at the higher level and are reported in Table 1 (see Supporting Information for the full table of high-level calculation results). The entries in Table 1 can be analyzed to look for general rules that may explain their small reorganization energy.

**Table 1:** Results of High-Level Calculation (**Layer iii**) compared to **Layer ii** for 13 Representative Molecules

	Δ*E*_S1_^(low)^/eV	Δ*E*_S1_^(high)^/eV	λ_4p_^(low)^/meV	λ_4p_^(high)^/meV	BOD^(high)^
WEPGUU04	3.08	2.99	49.8	47.8	0.040
CIVNON	3.08	3.00	51.7	59.0	0.038
IGUTIO	3.62	3.57	57.1	55.2	0.085
VIFSEI	2.07	2.11	43.3	46.9	0.057
QALLOH	1.88	1.89	46.5	43.8	0.024
CITCUG	2.85	2.78	72.6	73.6	0.100
CITCOA	3.20	3.11	100.4	89.1	0.096
OPOPOZ	1.39	1.35	25.9	23.6	0.026
PHTHCY01	2.21	2.12	41.7	40.0	-a
QIQSER	1.56	1.51	45.2	49.6	0.049
WOJWIE	2.45	2.41	45.9	50.4	0.038
MUKKAE	3.11	2.99	78.5	78.2	0.174
PUTCEM	3.10	3.19	88.0	107.8	0.078

a S_1_ of PHTHCY01 is not the HOMO → LUMO excitation.

For BODIPY species, we hypothesize from visual inspection that the small BOD and reorganization energy comes from the similarity of HOMO and LUMO at the two symmetric ends of the molecules (with opposite phases) and the nonbonding character of LUMO orbital in the central part of the molecule. These characteristics explain a very similar bond order in HOMOs and LUMOs and are shown in Figure 5a. Similar features can also be found in apparently unrelated molecules (e.g., VIFSEI and QALLOH in Figure 5, parts b and c, and QIQSER and WOJWIE (in Supporting Information), revealing that there may be a common mechanism that keeps the reorganization energy low in these cases. In the figure, we have indicated by A1 and A2 the two ends of the molecule with similar bonding patterns in HOMO and LUMO. The central part of the molecule, indicated as B, contains single atoms or small (4–5 membered) rings—reducing its ability to deform upon excitation. To demonstrate that the mechanism can be used to design new molecules, we create a model system with the A1–B–A2 structure, shown in Figure 5d, and displaying a small reorganization energy (69.7 meV) at the same level of theory. To be more quantitative, we reported in Figure 5 the overlap between HOMO and LUMO orbitals only considering the block of atoms in portion A1 or A2 and compared it with the HOMO–HOMO and LUMO–LUMO overlap within the same portion. Except for the sign, they have similar values; i.e., the shapes of HOMO and LUMO orbitals at the two sides of the molecules are similar.

**Figure 5:**
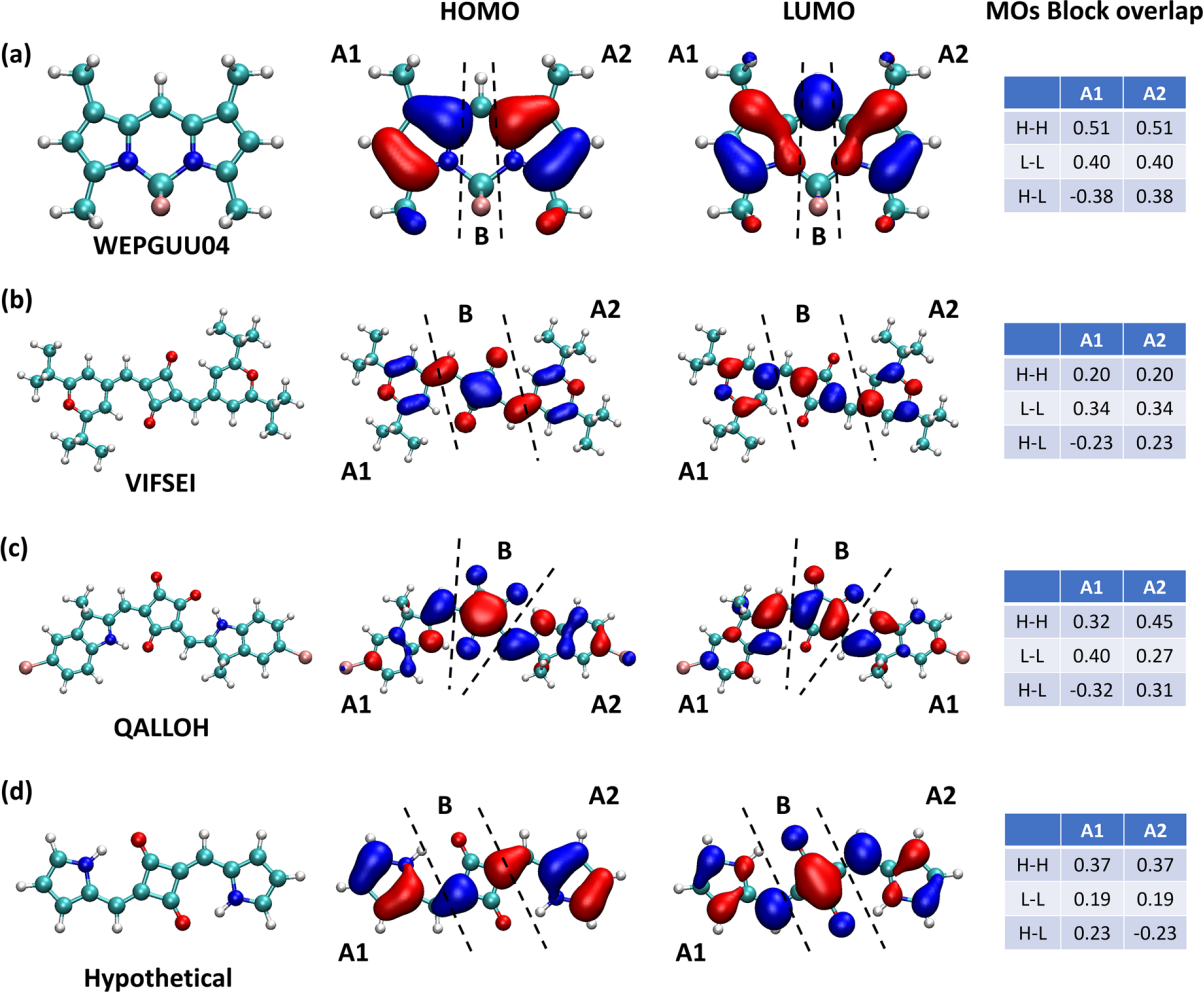
Analysis for A1–B–A2 systems for (a) WEPGUU04, (b) VIFSEI, (c) QALLOH, and (d) the model system. The 2nd/3rd columns show HOMOs and LUMOs plots with the definition of blocks for each system. The last column shows block overlaps among MOs (“H” for HOMO and “L” for LUMO).

The molecule CITGUG (and its 15 analogues) are polyenes with donor and acceptor groups at the two ends of the molecule. To describe the origin of the low reorganization energy, we consider a simple hexatriene denoted as **M1**, and the versions where one (two) donor(s) (−NH_2_) and acceptor(s) (−CN) replace the H at the end of the molecule, denoted as **M2** (**M3**). As shown in Figure 6, increasing the number of donors/acceptors causes a remarkable decrease in reorganization energy and BOD which can be associated with the increased nonbonding characters of the HOMO and LUMO orbitals (the figure also shows how the HOMO–LUMO gap and the excitation energy decrease in this case). The results are similar to the design idea of multiresonance TADF materials,^45^ i.e., minimizing bonding/antibonding feature in HOMO and LUMO, with the associated increase of nonbonding character, reducing the reorganization energy of molecules.

**Figure 6:**
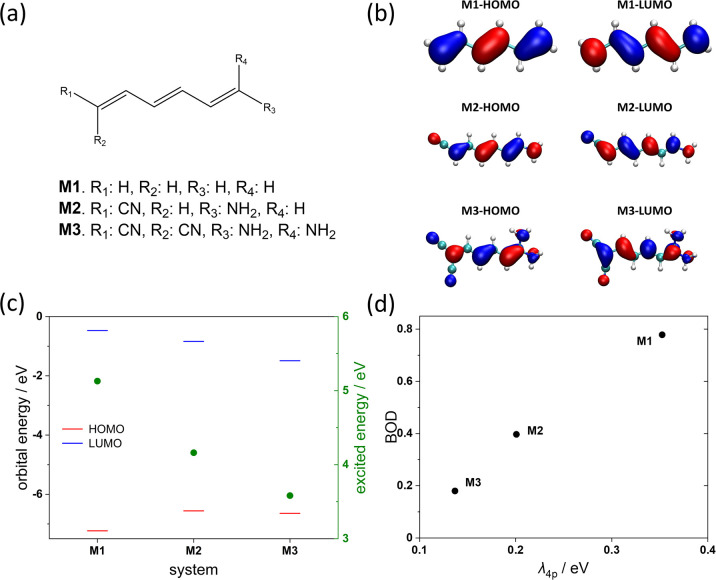
Model D–conjugate-A systems. (a) The geometries of model systems **M1**–**M3**. (b) Plots of HOMO/LUMO. (c) Orbital energies of HOMO and LUMO and excited energies of S_1_ state. (d) Relationship between reorganization energy and BOD. The calculation level is the same as in **layer iii**.

Overall, we could achieve a fairly good understanding of the overall mechanisms generally available to decrease the exciton reorganization energy. A possible exception that may require further investigation is MUKKAE, which displays unusually large BOD associated (see Supporting Information, Figure S25, for a possible explanation). Finally, we should also note that some important effects are best captured with a set of molecules designed ad hoc, which are difficult to analyze based on the large data set in our case. For example, Pi-Tai Chou et al.^33^ found that molecular reflection symmetry plays an important role in reducing reorganization energy in linear cyanine systems, while we have very few symmetric molecules.

In conclusion, a computational funnel approach was used in this work to identify molecules with small exciton reorganization energy and explain the origin of this feature. We have found that an approximate computational method based on a small basis set and single-point calculations of the force in the excited state is very effective for this type of screening. The calculations were used to look for orbital properties that can be used to identify, even more rapidly, molecules with potentially small exciton reorganization energies without performing excited state calculations. We have found that the delocalization of frontier orbitals correlates poorly with exciton reorganization energy, but a good prediction based on the ground state orbitals can be performed using the total bond order difference between the ground state and the HOMO → LUMO single excited configuration. The molecules with small reorganization energy have been grouped according to chemical similarity, and representatives of each group have been computed at a higher level of theory to validate the predictions and identify common patterns among them. Two quite different cases appeared to be frequent (i) molecules with similar bonding/antibonding patterns in HOMO and LUMO (often A–B–A symmetric molecules) and (ii) molecules with a strong nonbonding character of *both* HOMO and LUMO (linear polyenes with donor and acceptor substituents). This work highlights how high-throughput methods can be used at the same time to identify molecules with interesting characteristics, establish statistically robust correlations, and provide novel insights or design rules.

## Supporting Information Available

Results of preliminary investigation, general relation between reorganization energy and other parameters, detailed information on grouping, and accurate electronic structure results of representative molecules (PDF)
Transparent Peer Review report available (PDF)

## References

[ref1] Geffroy B.; Le Roy P.; Prat C. Organic light-emitting diode (OLED) technology: materials, devices and display technologies. Polym. Int. 2006, 55 (6), 572–582. 10.1002/pi.1974.

[ref2] Tao Y.; Yuan K.; Chen T.; Xu P.; Li H.; Chen R.; Zheng C.; Zhang L.; Huang W. Thermally Activated Delayed Fluorescence Materials Towards the Breakthrough of Organoelectronics. Adv. Mater. 2014, 26 (47), 7931–7958. 10.1002/adma.201402532. 25230116

[ref3] Jou J.-H.; Kumar S.; Agrawal A.; Li T.-H.; Sahoo S. Approaches for fabricating high efficiency organic light emitting diodes. J. Mater. Chem. C 2015, 3 (13), 2974–3002. 10.1039/C4TC02495H.

[ref4] Eng J.; Penfold T. J. Understanding and Designing Thermally Activated Delayed Fluorescence Emitters: Beyond the Energy Gap Approximation. Chem. Rec. 2020, 20 (8), 831–856. 10.1002/tcr.202000013. 32267093

[ref5] Ahmad S. A.; Eng J.; Penfold T. J. Rapid predictions of the colour purity of luminescent organic molecules. J. Mater. Chem. C 2022, 10 (12), 4785–4794. 10.1039/D1TC04748E.

[ref6] Menke S. M.; Holmes R. J. Exciton diffusion in organic photovoltaic cells. Energy Environ. Sci. 2014, 7 (2), 499–512. 10.1039/C3EE42444H.

[ref7] Tamai Y.; Ohkita H.; Benten H.; Ito S. Exciton Diffusion in Conjugated Polymers: From Fundamental Understanding to Improvement in Photovoltaic Conversion Efficiency. J. Phys. Chem. Lett. 2015, 6 (17), 3417–3428. 10.1021/acs.jpclett.5b01147. 26269208

[ref8] Mikhnenko O. V.; Blom P. W. M.; Nguyen T.-Q. Exciton diffusion in organic semiconductors. Energy Environ. Sci. 2015, 8 (7), 1867–1888. 10.1039/C5EE00925A.

[ref9] Sharma A.; Zhang L.; Tollerud J. O.; Dong M.; Zhu Y.; Halbich R.; Vogl T.; Liang K.; Nguyen H. T.; Wang F.; et al. Supertransport of excitons in atomically thin organic semiconductors at the 2D quantum limit. Light Sci. Appl. 2020, 9 (1), 116 10.1038/s41377-020-00347-y. 32655861 PMC7338549

[ref10] Aragó J.; Troisi A. Regimes of Exciton Transport in Molecular Crystals in the Presence of Dynamic Disorder. Adv. Funct. Mater. 2016, 26 (14), 2316–2325. 10.1002/adfm.201503888.

[ref11] Giannini S.; Peng W.-T.; Cupellini L.; Padula D.; Carof A.; Blumberger J. Exciton transport in molecular organic semiconductors boosted by transient quantum delocalization. Nat. Commun. 2022, 13 (1), 2755 10.1038/s41467-022-30308-5. 35589694 PMC9120088

[ref12] Mishra A.; Popovic D.; Vogt A.; Kast H.; Leitner T.; Walzer K.; Pfeiffer M.; Mena-Osteritz E.; Bäuerle P. A-D-A-type *S*,*N*-Heteropentacenes: Next-Generation Molecular Donor Materials for Efficient Vacuum-Processed Organic Solar Cells. Adv. Mater. 2014, 26 (42), 7217–7223. 10.1002/adma.201402448. 25244527

[ref13] Haedler A. T.; Kreger K.; Issac A.; Wittmann B.; Kivala M.; Hammer N.; Köhler J.; Schmidt H.-W.; Hildner R. Long-range energy transport in single supramolecular nanofibres at room temperature. Nature 2015, 523 (7559), 196–199. 10.1038/nature14570. 26156373

[ref14] Jin X.-H.; Price M. B.; Finnegan J. R.; Boott C. E.; Richter J. M.; Rao A.; Menke S. M.; Friend R. H.; Whittell G. R.; Manners I. Long-range exciton transport in conjugated polymer nanofibers prepared by seeded growth. Science 2018, 360 (6391), 897–900. 10.1126/science.aar8104. 29798881

[ref15] Sneyd A. J.; Beljonne D.; Rao A. A New Frontier in Exciton Transport: Transient Delocalization. J. Phys. Chem. Lett. 2022, 13 (29), 6820–6830. 10.1021/acs.jpclett.2c01133. 35857739 PMC9340810

[ref16] Padula D.; Omar Ö. H.; Nematiaram T.; Troisi A. Singlet fission molecules among known compounds: finding a few needles in a haystack. Energy Environ. Sci. 2019, 12 (8), 2412–2416. 10.1039/C9EE01508F.

[ref17] Zhao K.; Omar Ö. H.; Nematiaram T.; Padula D.; Troisi A. Novel thermally activated delayed fluorescence materials by high-throughput virtual screening: going beyond donor–acceptor design. J. Mater. Chem. C 2021, 9 (9), 3324–3333. 10.1039/D1TC00002K.

[ref18] Lopez S. A.; Sanchez-Lengeling B.; de Goes Soares J.; Aspuru-Guzik A. Design principles and top non-fullerene acceptor candidates for organic photovoltaics. Joule 2017, 1 (4), 857–870. 10.1016/j.joule.2017.10.006.

[ref19] Zhao Z.-W.; Omar Ö. H.; Padula D.; Geng Y.; Troisi A. Computational Identification of Novel Families of Nonfullerene Acceptors by Modification of Known Compounds. J. Phys. Chem. Lett. 2021, 12 (20), 5009–5015. 10.1021/acs.jpclett.1c01010. 34018746

[ref20] Padula D.; Lee M. H.; Claridge K.; Troisi A. Chromophore-Dependent Intramolecular Exciton–Vibrational Coupling in the FMO Complex: Quantification and Importance for Exciton Dynamics. J. Phys. Chem. B 2017, 121 (43), 10026–10035. 10.1021/acs.jpcb.7b08020. 28990788

[ref21] Hazra A.; Chang H. H.; Nooijen M. First principles simulation of the UV absorption spectrum of ethylene using the vertical Franck-Condon approach. J. Chem. Phys. 2004, 121 (5), 2125–2136. 10.1063/1.1768173. 15260766

[ref22] Avila Ferrer F. J.; Santoro F. Comparison of vertical and adiabatic harmonic approaches for the calculation of the vibrational structure of electronic spectra. Phys. Chem. Chem. Phys. 2012, 14 (39), 13549 10.1039/c2cp41169e. 22847219

[ref23] Omar Ö. H.; Nematiaram T.; Troisi A.; Padula D. Organic materials repurposing, a data set for theoretical predictions of new applications for existing compounds. Sci. Data 2022, 9 (1), 54 10.1038/s41597-022-01142-7. 35165288 PMC8844419

[ref24] Groom C. R.; Bruno I. J.; Lightfoot M. P.; Ward S. C. The Cambridge Structural Database. Acta. Crystallogr. B. Struct. 2016, 72 (2), 171–179. 10.1107/S2052520616003954. PMC4822653 27048719

[ref25] Aragó J.; Troisi A. Dynamics of the Excitonic Coupling in Organic Crystals. Phys. Rev. Lett. 2015, 114 (2), 026402 10.1103/PhysRevLett.114.026402. 25635554

[ref26] Xie X.; Santana-Bonilla A.; Troisi A. Nonlocal Electron–Phonon Coupling in Prototypical Molecular Semiconductors from First Principles. J. Chem. Theory Comput. 2018, 14 (7), 3752–3762. 10.1021/acs.jctc.8b00235. 29851487

[ref27] Gaussian 16, Rev. A.03; Gaussian Inc.: Wallingford, CT, 2016.

[ref28] Xie X.; Troisi A. Supporting data for this work; 2023. https://github.com/XiaoyuUoL/ForceApproach (accessed 13 March, 2023 ).

[ref29] Stehr V.; Fink R. F.; Tafipolski M.; Deibel C.; Engels B. Comparison of different rate constant expressions for the prediction of charge and energy transport in oligoacenes. WIREs Comput. Mol. Sci. 2016, 6 (6), 694–720. 10.1002/wcms.1273.

[ref30] Mayer I. Charge, bond order and valence in the AB initio SCF theory. Chem. Phys. Lett. 1983, 97 (3), 270–274. 10.1016/0009-2614(83)80005-0.

[ref31] Mayer I. Bond orders and valences from ab initio wave functions. Int. J. Quantum Chem. 1986, 29 (3), 477–483. 10.1002/qua.560290320.

[ref32] Mayer I. On bond orders and valences in the Ab initio quantum chemical theory. Int. J. Quantum Chem. 1986, 29 (1), 73–84. 10.1002/qua.560290108.

[ref33] Wu C.-C.; Li E. Y.; Chou P.-T. Reducing the internal reorganization energy via symmetry controlled π-electron delocalization. Chem. Sci. 2022, 13 (24), 7181–7189. 10.1039/D2SC01851A. 35799804 PMC9214956

[ref34] Shuai Z.; Geng H.; Xu W.; Liao Y.; André J.-M. From charge transport parameters to charge mobility in organic semiconductors through multiscale simulation. Chem. Soc. Rev. 2014, 43 (8), 2662 10.1039/c3cs60319a. 24394992

[ref35] Chen W.-C.; Cheng Y.-C. Elucidating the Magnitude of Internal Reorganization Energy of Molecular Excited States from the Perspective of Transition Density. J. Phys. Chem. A 2020, 124 (38), 7644–7657. 10.1021/acs.jpca.0c06482. 32864966

[ref36] Ai Q.; Bhat V.; Ryno S. M.; Jarolimek K.; Sornberger P.; Smith A.; Haley M. M.; Anthony J. E.; Risko C. OCELOT: An infrastructure for data-driven research to discover and design crystalline organic semiconductors. J. Chem. Phys. 2021, 154 (17), 174705 10.1063/5.0048714. 34241085

[ref37] Gallarati S.; Van Gerwen P.; Laplaza R.; Vela S.; Fabrizio A.; Corminboeuf C. OSCAR: an extensive repository of chemically and functionally diverse organocatalysts. Chem. Sci. 2022, 13 (46), 13782–13794. 10.1039/D2SC04251G. 36544722 PMC9710326

[ref38] Loudet A.; Burgess K. BODIPY Dyes and Their Derivatives: Syntheses and Spectroscopic Properties. Chem. Rev. 2007, 107 (11), 4891–4932. 10.1021/cr078381n. 17924696

[ref39] Ulrich G.; Ziessel R.; Harriman A. The Chemistry of Fluorescent Bodipy Dyes: Versatility Unsurpassed. Angew. Chem., Int. Ed. 2008, 47 (7), 1184–1201. 10.1002/anie.200702070. 18092309

[ref40] Fischer G. M.; Isomäki-Krondahl M.; Göttker-Schnetmann I.; Daltrozzo E.; Zumbusch A. Pyrrolopyrrole Cyanine Dyes: A New Class of Near-Infrared Dyes and Fluorophores. Chem. Eur. J. 2009, 15 (19), 4857–4864. 10.1002/chem.200801996. 19296481

[ref41] Peceli D.; Hu H.; Fishman D. A.; Webster S.; Przhonska O. V.; Kurdyukov V. V.; Slominsky Y. L.; Tolmachev A. I.; Kachkovski A. D.; Gerasov A. O.; et al. Enhanced Intersystem Crossing Rate in Polymethine-Like Molecules: Sulfur-Containing Squaraines versus Oxygen-Containing Analogues. J. Phys. Chem. A 2013, 117 (11), 2333–2346. 10.1021/jp400276g. 23427868

[ref42] Mayerhöffer U.; Gsänger M.; Stolte M.; Fimmel B.; Würthner F. Synthesis and Molecular Properties of Acceptor-Substituted Squaraine Dyes. Chem. Eur. J. 2013, 19 (1), 218–232. 10.1002/chem.201202783. 23180571

[ref43] Punzi A.; Capozzi M. A. M.; Fino V.; Carlucci C.; Suriano M.; Mesto E.; Schingaro E.; Orgiu E.; Bonacchi S.; Leydecker T.; et al. Croconaines as molecular materials for organic electronics: synthesis, solid state structure and use in transistor devices. J. Mater. Chem. C 2016, 4 (15), 3138–3142. 10.1039/C6TC00264A.

[ref44] Maeda T.; Oka T.; Sakamaki D.; Fujiwara H.; Suzuki N.; Yagi S.; Konishi T.; Kamada K. Unveiling a new aspect of oxocarbons: open-shell character of 4- and 5-membered oxocarbon derivatives showing near-infrared absorption. Chem. Sci. 2023, 14, 1978 10.1039/D2SC06612B. 36845939 PMC9944335

[ref45] Hatakeyama T.; Shiren K.; Nakajima K.; Nomura S.; Nakatsuka S.; Kinoshita K.; Ni J.; Ono Y.; Ikuta T. Ultrapure Blue Thermally Activated Delayed Fluorescence Molecules: Efficient HOMO-LUMO Separation by the Multiple Resonance Effect. Adv. Mater. 2016, 28 (14), 2777–2781. 10.1002/adma.201505491. 26865384

