# Supplementary material for: Identification via Virtual Screening of Emissive Molecules with a Small Exciton–Vibration Coupling for High Color Purity and Potential Large Exciton Delocalization
Source: J Phys Chem Lett. 2023 Apr 27;14(17):4119–26. doi: 10.1021/acs.jpclett.3c00749 (PMC10165648; doi:10.1021/acs.jpclett.3c00749)
Supplement: Supplementary file 1 — jz3c00749_si_001.pdf [file jz3c00749_si_001.pdf]

Supporting Information

Identification via Virtual Screening of Emissive Molecules with Small Exciton-Vibration Coupling for High Colour Purity and Potential Large Exciton Delocalisation

Xiaoyu Xie*, Alessandro Troisi*

Department of Chemistry, University of Liverpool L69 3BX, UK

* e-mail: xiaoyu@liverpool.ac.uk, a.troisi@liverpool.ac.uk

Repository[1] contains the following data:

(i) Table of CSD ID and λ_{force} for 4476 molecules (**layer (i)**)

(ii) Table of CSD ID with λ_{force} and λ_{4p} for 282 molecules computed at level **layer (ii)** and their grouping information

(iii) Ground and excited state optimized geometry for 282 molecules (**layer (ii)**)

(iv) Ground and excited state optimized geometry for 17 molecules computed at level **layer (iii)**

(v) ZINC ID, SMILES, λ_{force}, λ_{4p} and ground state geometry for the 391 molecules used in the preliminary study shown in **Figure 2** of the main manuscript.

S1. Preliminary calculation

We built two preliminary testing datasets before the screening works in the main text to validate the force approach and test the performance of calculation parameters such as basis set and optimization options. For convenience, S_0 optimization, S_1 optimization, S_0 frequency analysis and S_1 force calculation are labelled 'S_0Opt', 'S_1Opt', 'S_0Freq' and 'S_1Force', respectively.

S1.1. Validation of the force approach

To evaluate the time cost and accuracy of the force approach, 391 organic molecules (< 50 atoms, C/H/O/N elements) randomly selected from the ZINC dataset are calculated using the two approaches with B3LYP/6-31g(d) calculation level. The data for this test are included in the repository [1]. The time cost is displayed in **Figure S1,** as shown below.

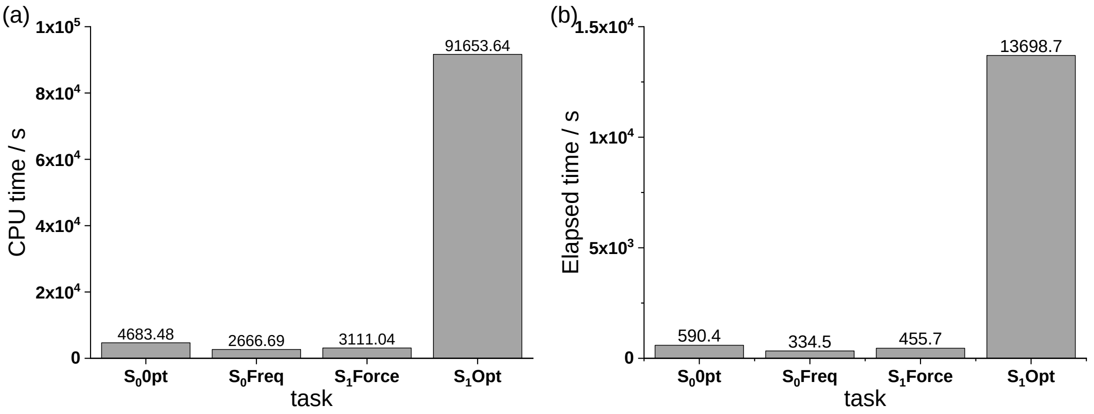

Figure S1. (a) CPU and (b) elapsed time cost of different DFT/TDDFT calculation tasks. (4-core is used in this testing)

S1.2. Basis set and optimization options

50 median-size organic molecules (20~25 atoms) consisting of C/H/O/N elements, randomly selected from the ZINC dataset, are calculated using two different calculation levels (M06-2X/def2-SVP and M06-2X/3-21g*), and 'opt=default' and 'opt=loose' options are tested for the two optimizations tasks ('S_0Opt' and 'S_1Opt').

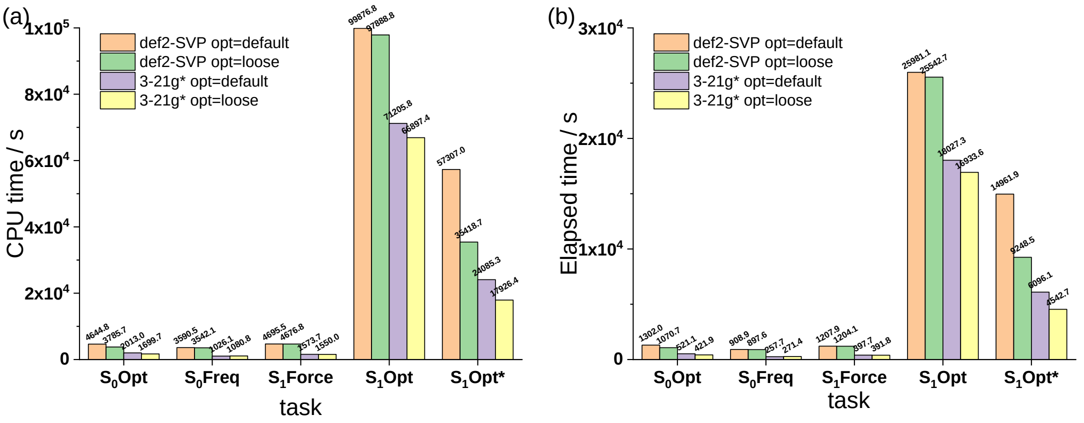

Figure S2. (a) CPU and (b) elapsed time cost of different DFT/TDDFT calculation tasks. (4-core is used in this testing). 'S1Opt*' shows the average time cost by removing error S_1 optimizations (6 and 10 in 50 molecules for def2-SVP and 3-21g*, respectively).

Figure S2 lists the CPU and elapsed time costs of 4-core DFT/TDDFT calculations. Here are some conclusions:

[1]. 'S_1Opt' is the most time-consuming task, which can be avoided in our approach, while the other three tasks take comparable average time costs. Considering the average time cost of each task, 0.94/0.32 h per molecule is needed for our approach if the def2-SVP/3-21g* basis set is applied with a 4-core parallel.

[2] Some 'S_1opt' calculations fail for several reasons (six systems for def2-SVP basis set and ten systems for 3-21g*). e.g., degenerate S_0/S_1 during the S_1 optimization (i.e., canonical interaction point), unconverged S_1 excited energy, etc. All these failed tasks can be terminated during the calculation process (based on the optimization cycles and time cost). In **Figure S3**, we display the average time-cost ('S_1opt*') by removing the failed S1opt with CPU time cost larger than 400000/100000 s for the def2-SVP and 3-21g*, respectively.

[3]. The def2-SVP basis set costs about three times of 3-21g* basis set for single point calculations ('S_0Freq' and 'S_1Force') and two times for optimization tasks ('S_0Opt' and 'S_1Opt').

[4]. By reducing the convergence criterion (adding the 'opt=loose' option), time can be saved for both S_0 and S_1 optimization tasks, especially for S_1 optimization when manual termination is performed. (i.e., 'S_1opt*') Moreover, as shown in **Figure S3**, we also plot reorganization energies (λ_{4p} and λ_{force}) calculated via different calculation levels, 'opt=loose' does not affect the results much.

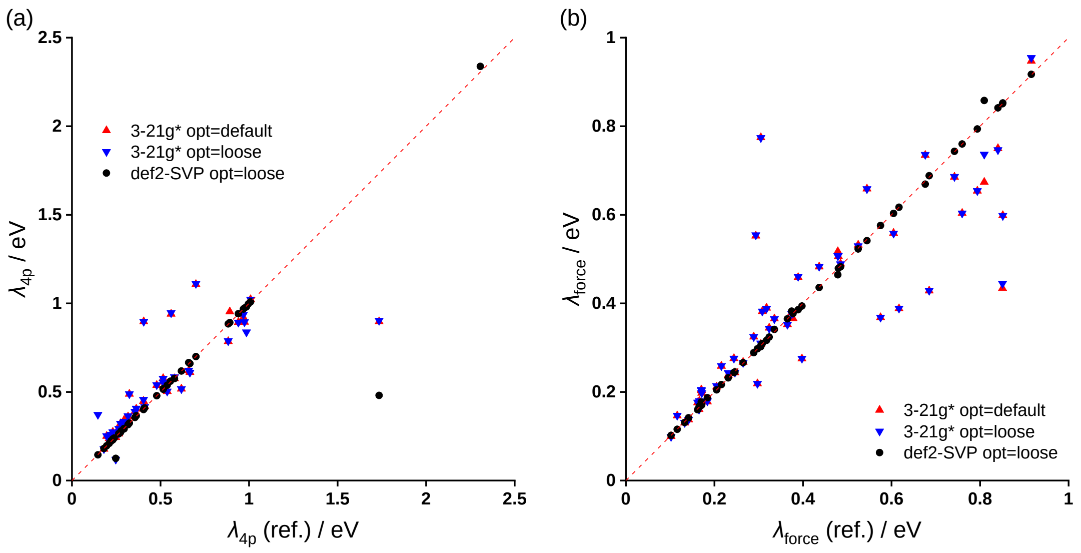

Figure S3. Comparison of calculated reorganization energies via (a) equation 1 (λ_{4p}) and (b) equation 3 (λ_{force}) in the main text with different calculation levels and optimization options. Here, the def2-SVP basis set with 'opt=default' is the reference.

Table S1. Summary of time costs and accuracy of using a small basis set.

Basis set		Def2-SVP (Ref)	Def2-SVP	3-21g*	3-21g*
If 'opt=loose'		No	Yes	No	Yes
Time cost / h	S_0Opt	1.29 (0.36)	1.05 (0.30)	0.56 (0.14)	0.47 (0.12)
	S_0Freq	0.99 (0.25)		0.30 (0.08)	
	S_1Force	1.30 (0.34)		0.44 (0.11)	
CPU (Elapsed)	S_1Opt	27.74 (7.22)	27.19 (7.10)	19.78 (5.01)	18.58 (4.70)
	S_1Opt*	15.92 (4.16)	9.84 (2.57)	6.69 (1.69)	4.98 (1.26)
R of λ_{4p}		-	0.89	0.81	0.81
R of λ_{force}		-	0.99	0.83	0.83

S2. Additional information on calculation results

S2.1. Results of the reorganization energy

In the main text, the correlation between reorganization energy and two properties is discussed. One is delocalization. We assume that HOMO to LUMO excitation is dominant for S_1 and use the average inverse participation ratio (IPR) of HOMO and LUMO to evaluate the delocalization of S_1. A molecular orbital (MO) $|\psi\rangle$ can be expressed as a linear combination of the original atomic orbitals (AO) $|\phi_i^0\rangle$,

$$|\psi\rangle = \sum_i c_i^0 |\phi_i^0\rangle,$$

Then, the IPR of the MO $|\psi\rangle$ can be defined as,

$$\mathrm{IPR} = \frac{\sum_a \sum_{i\in a} c_i^2}{\sum_a \left(\sum_{i\in a} c_i^2\right)^2} = \left(\sum_a \left(\sum_{i\in a} c_i^2\right)^2\right)^{-1}.$$

Here, a is an index of an atom, and c_i is the coefficient of an orthonormalized atomic orbital i with $\boldsymbol{c} = \boldsymbol{S}^{1/2}\boldsymbol{c}_0$ ($\boldsymbol{S}$ is the overlap of the original AO basis, i.e., $\boldsymbol{S}_{ij} = \langle\phi_i|\phi_j\rangle$).

The relationship between reorganization energy and averaged IPR is shown in **Figure S4**.

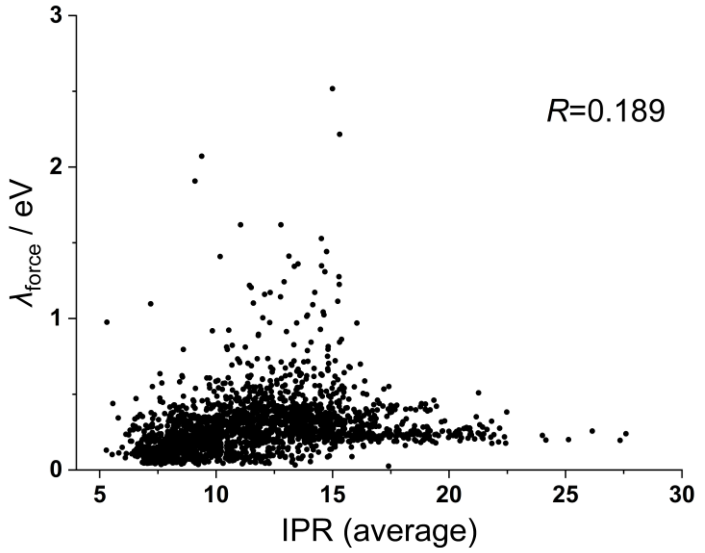

Figure S4. The correlation between delocalization (averaged IPR) and reorganization energy λ_{force} for molecules with the weight of HOMO to LUMO excitation larger than 0.95.

Another property is the bond order difference. In practice, we use the definition of Mayer's bond order [2-4], as shown below.

For a general wavefunction, the density matrix (AO basis) is defined as,

$$\boldsymbol{P}_{AO}^{(\alpha/\beta)} = \boldsymbol{C}\boldsymbol{P}_{MO}^{(\alpha/\beta)}\boldsymbol{C}^T.$$

And the total and spin density matrices,

$$\boldsymbol{P}_{AO} = \boldsymbol{P}_{AO}^{(\alpha)} + \boldsymbol{P}_{AO}^{(\beta)}, \boldsymbol{P}_{AO}^{(s)} = \boldsymbol{P}_{AO}^{(\alpha)} - \boldsymbol{P}_{AO}^{(\beta)}.$$

Here $\boldsymbol{C}$ is the MO coefficient matrix with respect to the original AO basis set of all MOs, and $P_{MO}^{(\alpha/\beta)}$ is the MO density matrix for α/β electrons. Then Mayer's bond order between atom a and b is defined as,

$$B_{ab} = 2\sum_{i\in a, j\in b}\left[\left(\boldsymbol{P}_{AO}^{(\alpha)}\boldsymbol{S}\right)_{ij}\left(\boldsymbol{P}_{AO}^{(\alpha)}\boldsymbol{S}\right)_{ji} + \left(\boldsymbol{P}_{AO}^{(\beta)}\boldsymbol{S}\right)_{ij}\left(\boldsymbol{P}_{AO}^{(\beta)}\boldsymbol{S}\right)_{ji}\right]$$
$$= \sum_{i\in a, j\in b}\left[(\boldsymbol{P}_{AO}\boldsymbol{S})_{ij}(\boldsymbol{P}_{AO}\boldsymbol{S})_{ji} + \left(\boldsymbol{P}_{AO}^{(s)}\boldsymbol{S}\right)_{ij}\left(\boldsymbol{P}_{AO}^{(s)}\boldsymbol{S}\right)_{ji}\right].$$

Where i and j are indices of AO orbitals, $\boldsymbol{S}$ is the AO overlap matrix.

For example, the MO density matrix of close-shell HF/DFT wavefunction is,

$$\boldsymbol{P}_{AO}^{(\alpha/\beta)} = \sum_{i\in occ} 1 * |i\rangle\langle i| = \boldsymbol{C}_{occ}\boldsymbol{C}_{ooc}^{T},$$

And

$$\boldsymbol{P}_{AO} = 2\boldsymbol{C}_{occ}\boldsymbol{C}_{ooc}^{T}.$$

Therefore,

$$B_{ab} = \sum_{i\in a, j\in b}(\boldsymbol{P}_{AO}\boldsymbol{S})_{ij}(\boldsymbol{P}_{AO}\boldsymbol{S})_{ji}.$$

Here, we display the statistical relationship between reorganization energy and BOD for all **layer (i)** molecules in **Figure S5**.

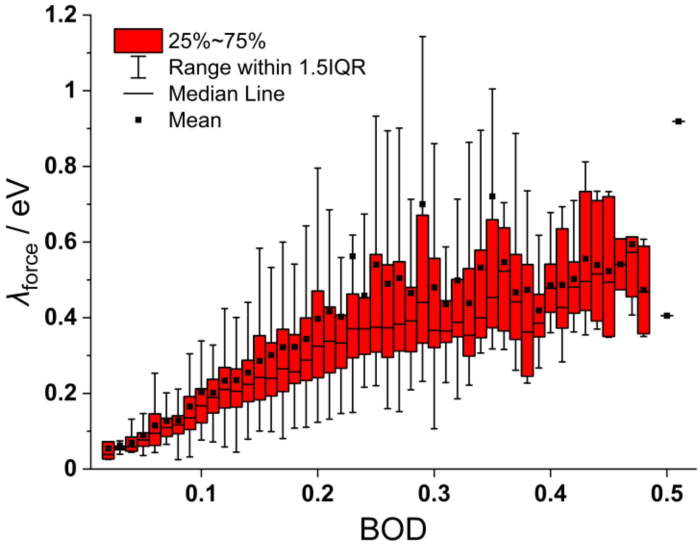

Figure S5. Boxplot that shows the range of expected λ_{force} for intervals of BOD for all molecules in **layer (i)**.

S2.2. Grouping: representative molecules and their analogous

Here we list 7 groups and 2 subgroups of BODIPY derivatives, display 2D draws of some molecules (as well as CSD ID and $\lambda_{4\mathrm{p}}$ results) in each group. For the full list, please check the repository.[1]

2.2.1. BODIPY species (196 molecules)

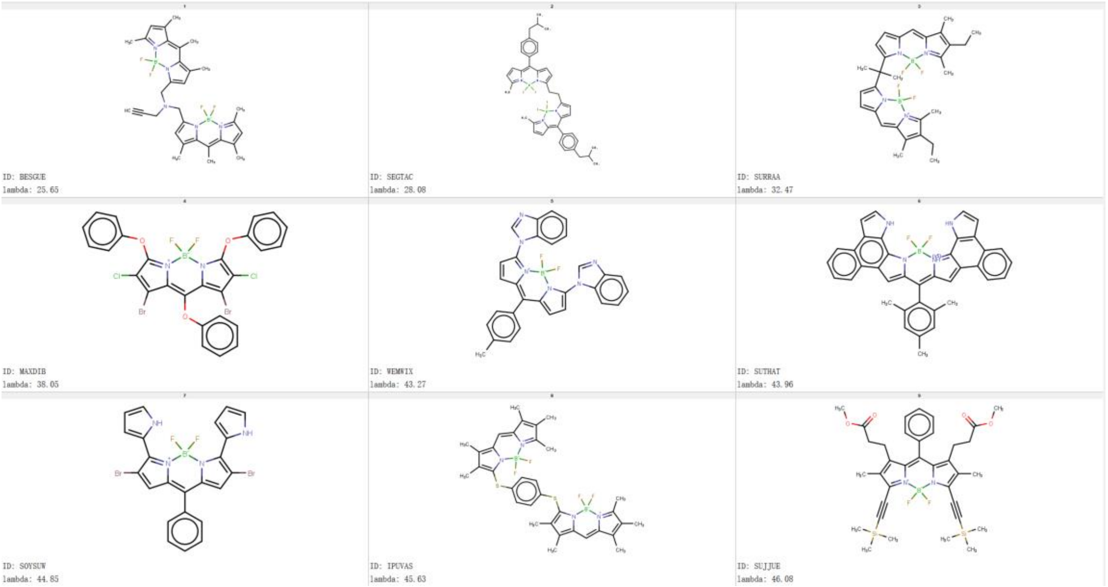

Figure S6. Nine molecules in set 1 (BODIPY species) with CSD ID and reorganization energy $\lambda_{4\mathrm{p}}$ (in meV).

2.2.1.1. BODIPY derivatives 1: change F elements (19 molecules)

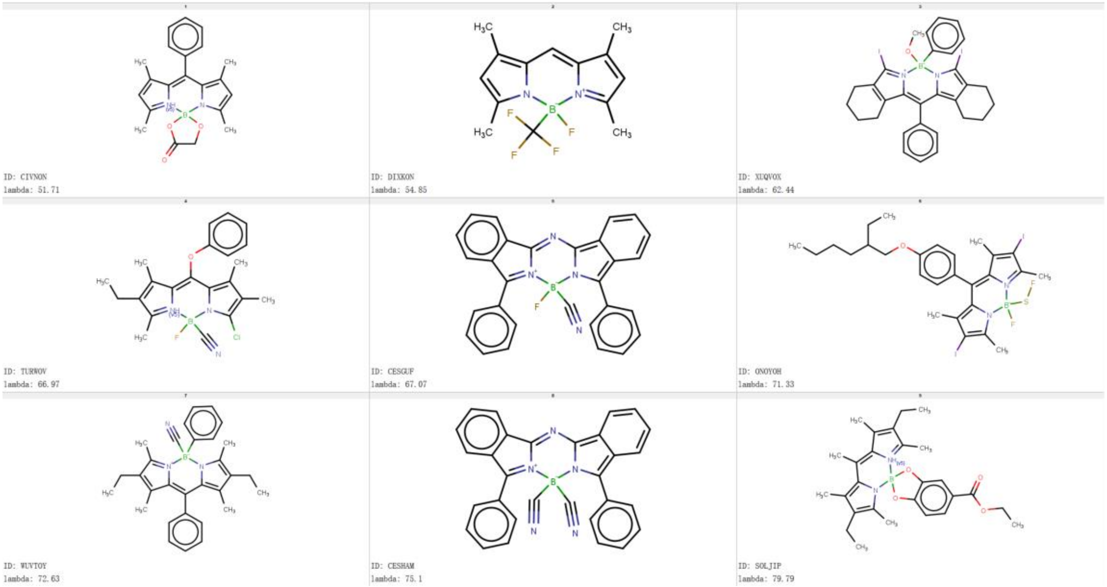

Figure S7. Nine molecules which are similar to CIVNON with CSD ID and reorganization energy $\lambda_{4\mathrm{p}}$ (in meV).

2.2.1.2 BODIPY derivatives 2: change N elements (22 molecules)

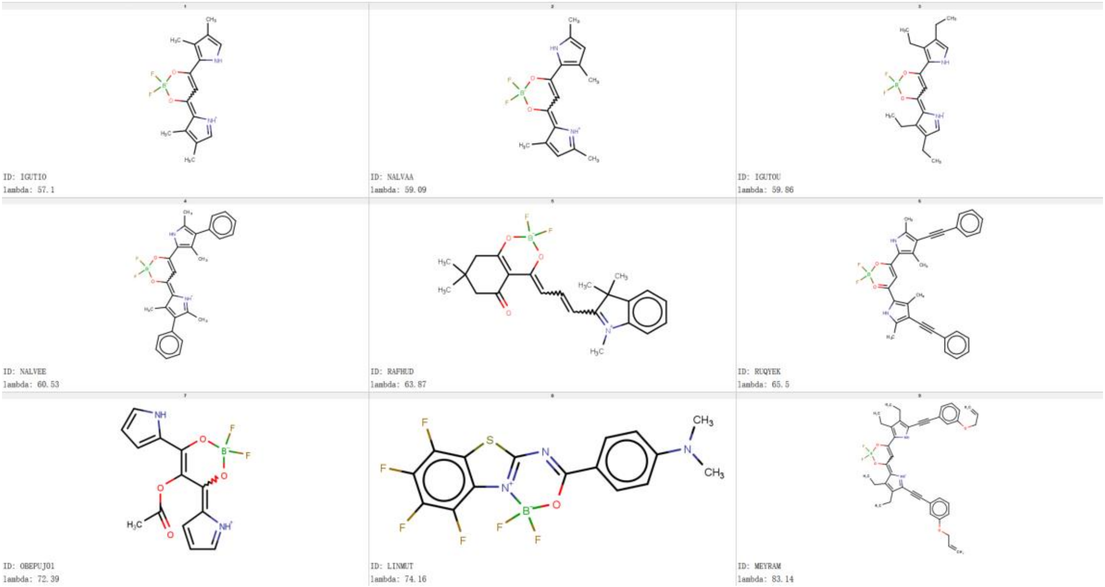

Figure S8. Nine molecules which are similar to IGUTIO with CSD ID and reorganization energy $\lambda_{4\mathrm{p}}$ (in meV).

2.2.2. Squaraine and croconaine dye (9 molecules)

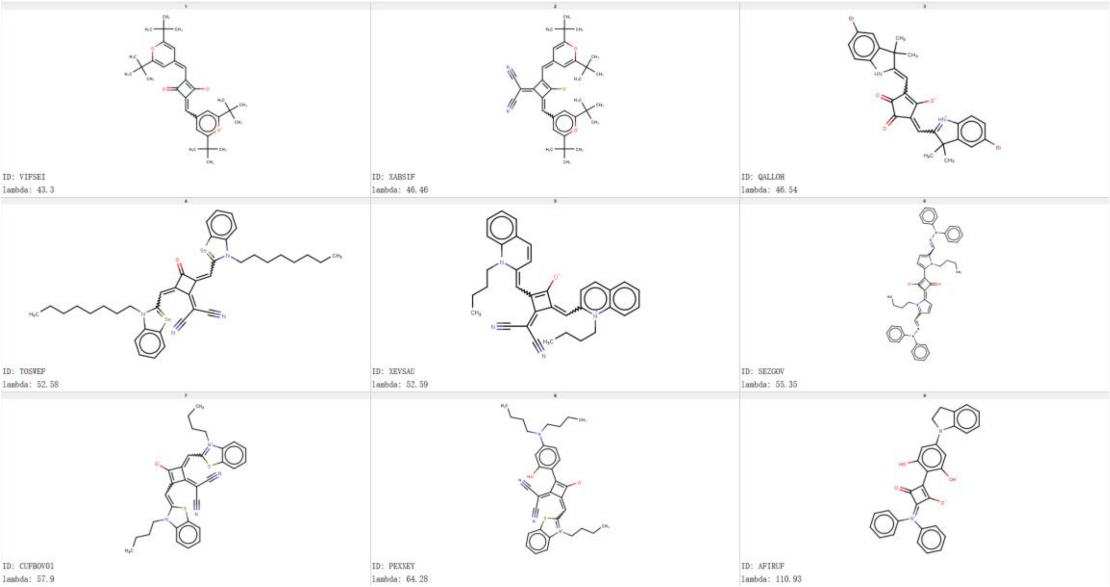

Figure S9. All Squaraine and croconaine dye molecules with CSD ID and reorganization energy $\lambda_{4\mathrm{p}}$ (in meV).

2.2.3. CITCUG and its analogous molecules (16 molecules)

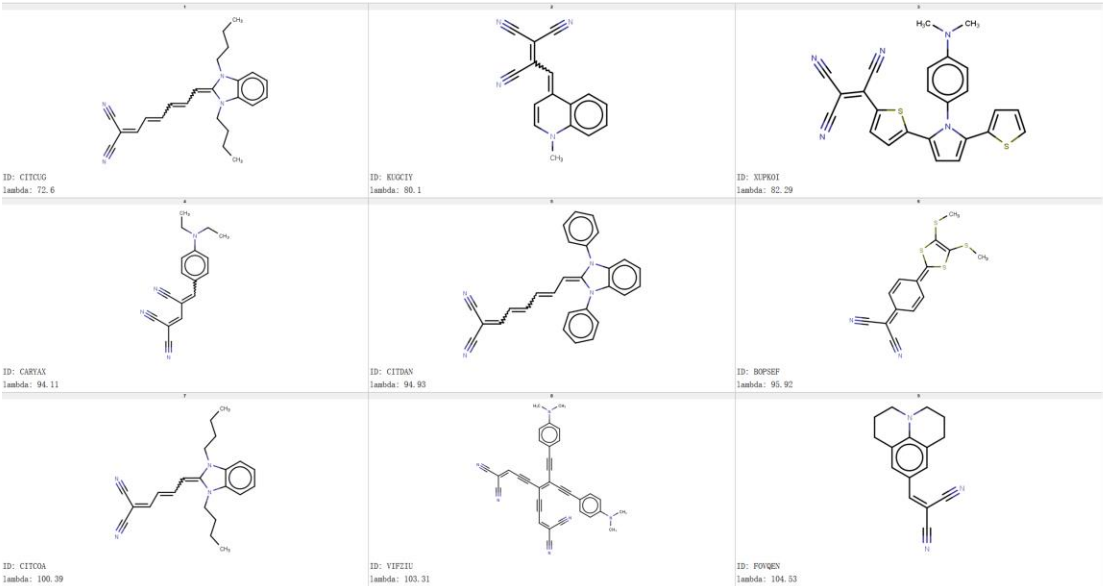

Figure S10. Nine molecules which are similar to CITCUG and CITCOA with CSD ID and reorganization energy λ_{4p} (in meV).

2.2.4. QIRQAJ and analogous molecules (6 molecules)

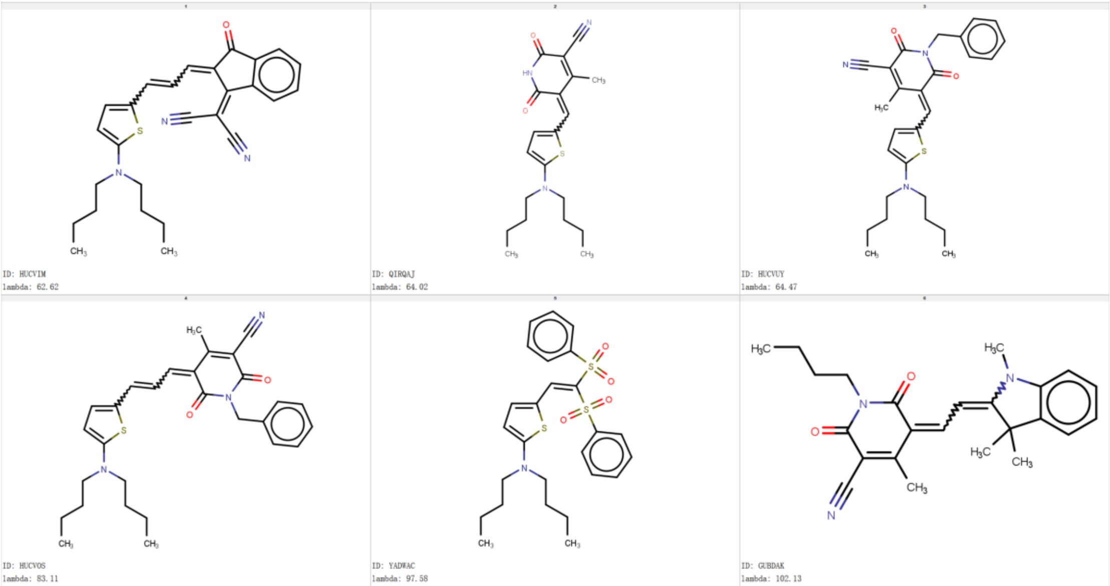

Figure S11. All molecules which are similar to QIRQAJ and GUBCAJ with CSD ID and reorganization energy λ_{4p} (in meV).

2.2.5. NELCUD and analogous molecules (2 molecules)

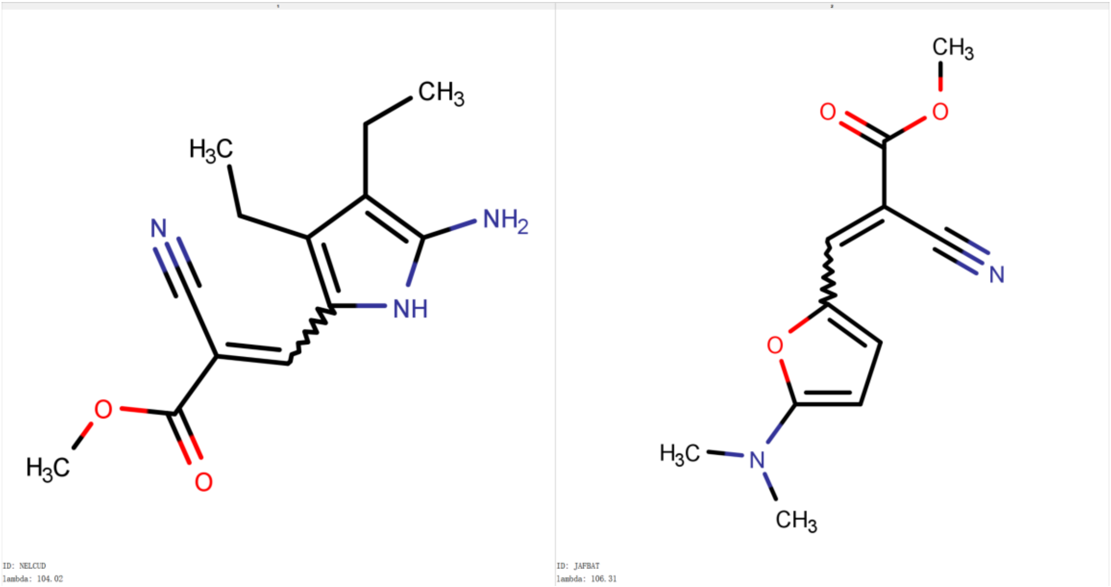

Figure S12. NELCUD and analogous molecule with CSD ID and reorganization energy λ_{4p} (in meV).

2.2.6. DOMZIO and analogous molecule (2 molecules)a

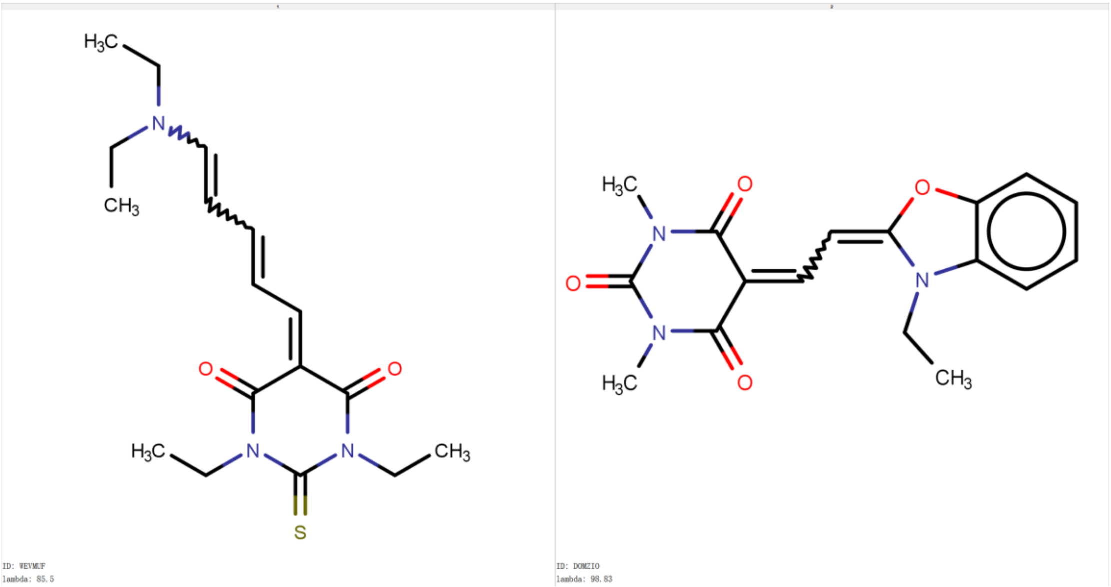

Figure S13. DOMZIO and analogous molecule with CSD ID and reorganization energy λ_{4p} (in meV).

2.2.7. Others (10 molecules)

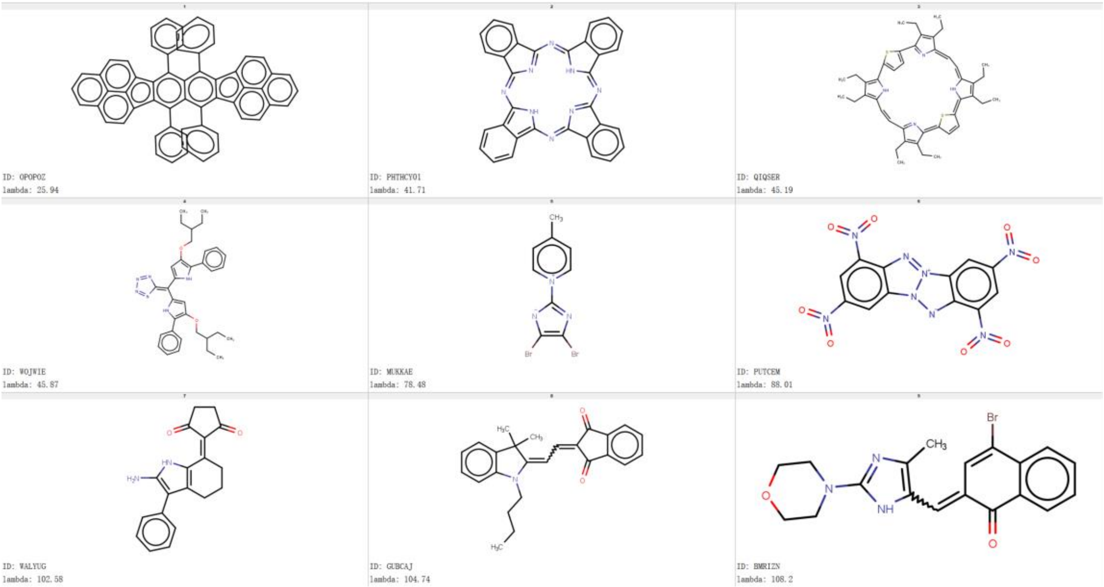

Figure S14. Nine molecules which do not have similar molecules with CSD ID and reorganization energy λ_{4p} (in meV).

S2.3. Full results of representative molecules

For each set in section S2.3, we select 1 or 2 molecules as representative molecules (6 molecules in set 7 with $\lambda < 100$ meV) for high-level calculations (**layer (iii)**). Table S2 lists the results of the high-level calculations compared to low-level ones (**layer (ii)**).

Table S2. Results of high-level calculation compared to low-level calculations.

CSD ID	$\Delta E_{\mathrm{S1}}^{(\mathrm{low})}$/eV	$\Delta E_{\mathrm{S1}}^{(\mathrm{high})}$/eV	$\lambda_{\mathrm{4p}}^{(\mathrm{low})}$/meV	$\lambda_{\mathrm{4p}}^{(\mathrm{high})}$/meV	BOD
WEPGUU04	3.08	2.99	49.8	47.8	0.040
CIVNON	3.08	3.00	51.7	59.0	0.038
IGUTIO	3.62	3.57	57.1	55.2	0.085
VIFSEI	2.07	2.11	43.3	46.8	0.057
QALLOH	1.88	1.89	46.5	43.8	0.073
CITCUG	2.85	2.78	72.6	73.6	0.100
CITCOA	3.20	3.11	100.4	89.1	0.096
QIRQAJ	3.04	3.02	64.0	-*	-
GUBCAJ	3.34	3.29	104.7	160.8	0.090
DOMZIO	3.68	3.59	98.8	126.4	0.083
NELCUD	3.67	3.67	104.0	186.7	0.102
OPOPOZ	1.39	1.35	25.9	23.6	0.026
PHTHCY01	2.21	2.12	41.7	40.0	-**
QIQSER	1.56	1.51	45.2	49.6	0.049
WOJWIE	2.45	2.41	45.9	50.4	0.038
MUKKAE	3.11	2.99	78.5	78.3	0.174
PUTCEM	3.10	3.19	88.0	107.9	0.078

* S_1 optimization fails. ** S_1 is not HOMO→LUMO excitation dominated.

Other results, including the difference in the bond length and the bond order between S_0 and S_1 and plots of HOMO and LUMO, are also displayed below.

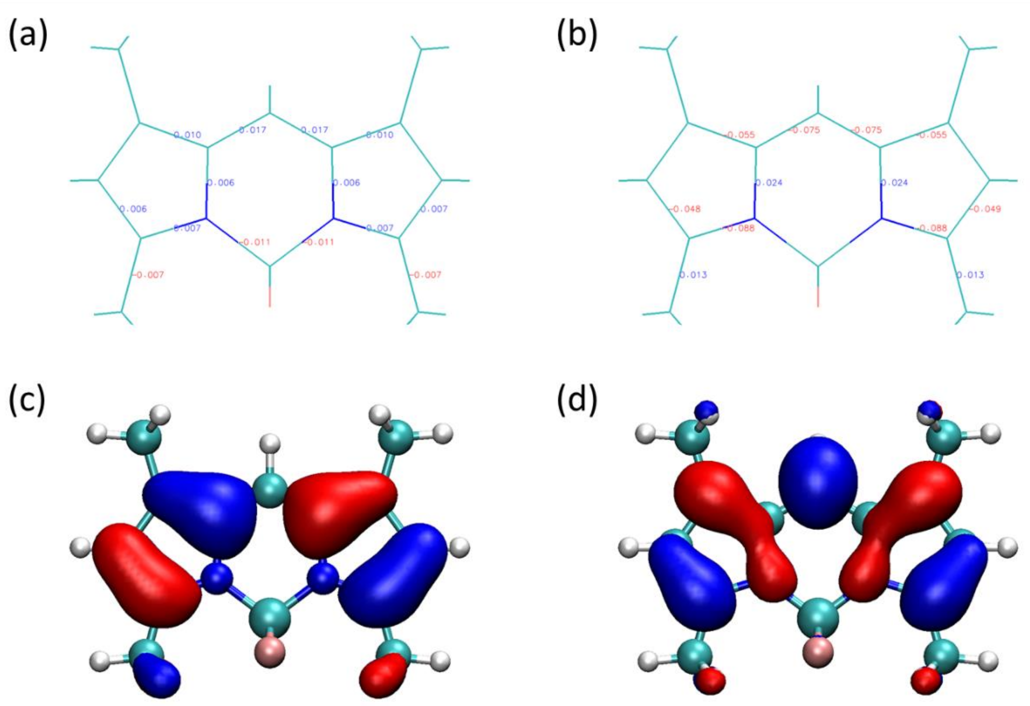

Figure S15. Results of high-level calculation for WEPGUU04. (a) Individual bond length difference (unit: Å) between S_0 and S_1 geometries. (b) Individual bond order difference between S0 state and HOMO-LUMO excitation state. (c) The plot of HOMO. (d) The plot of LUMO.

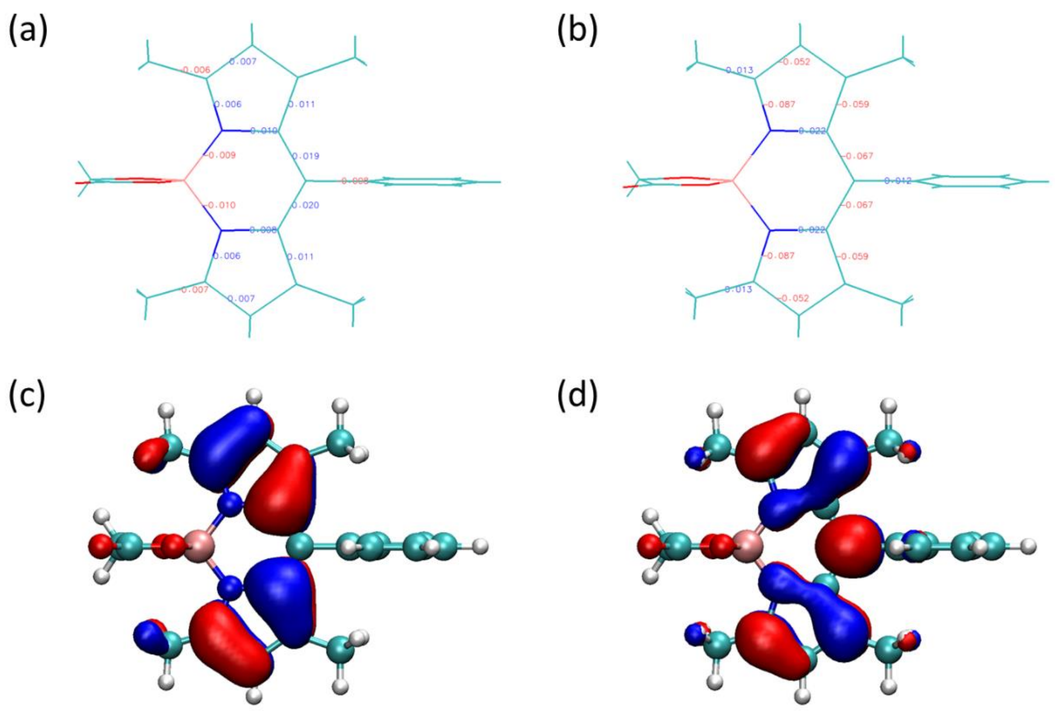

Figure S16. Results of high-level calculation for CIVNON. (a) Individual bond length difference (unit: Å) between S_0 and S_1 geometries. (b) Individual bond order difference between S0 state and HOMO-LUMO excitation state. (c) The plot of HOMO. (d) The plot of LUMO.

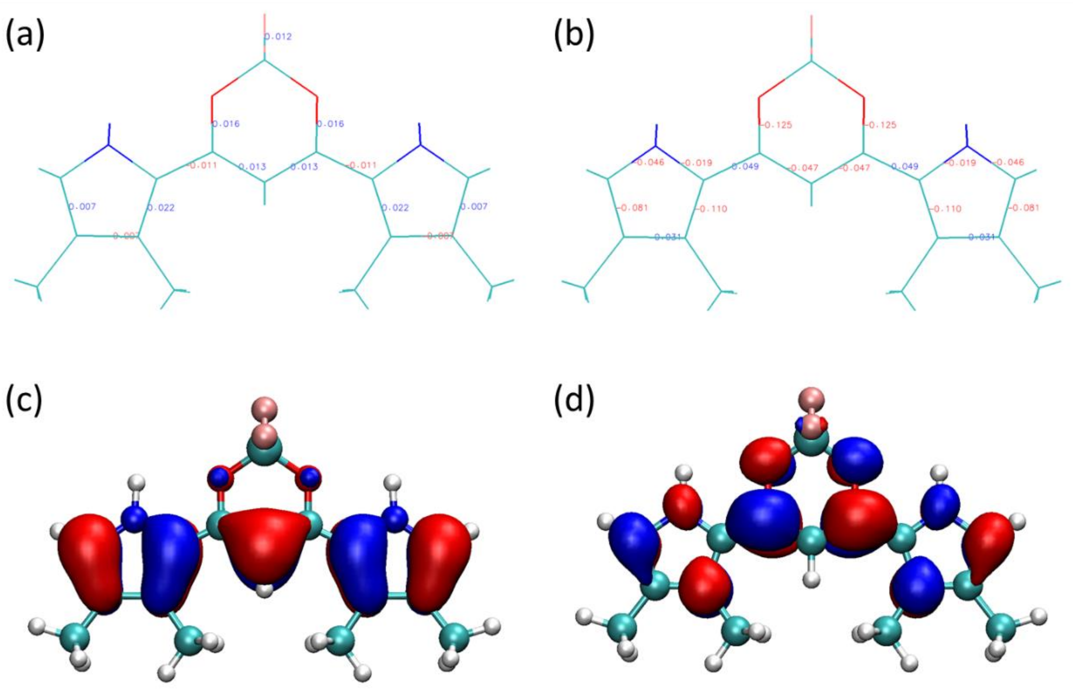

Figure S17. Results of high-level calculation for IGUTIO. (a) Individual bond length difference (unit: Å) between S_0 and S_1 geometries. (b) Individual bond order difference between S0 state and HOMO-LUMO excitation state. (c) The plot of HOMO. (d) The plot of LUMO.

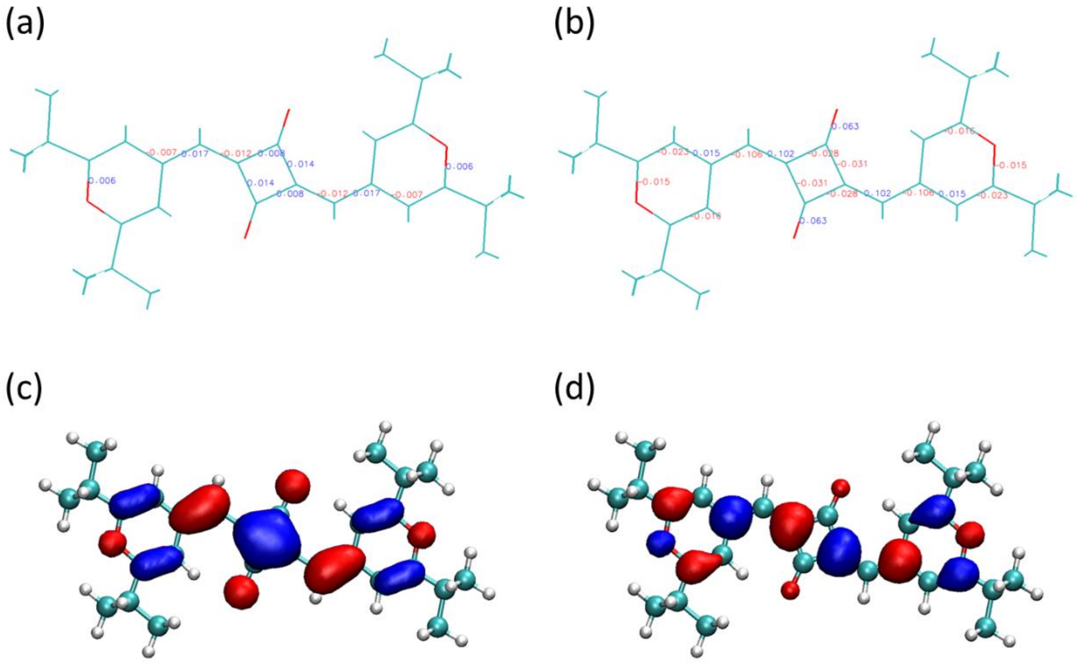

Figure S18. Results of high-level calculation for VIFSEI. (a) Individual bond length difference (unit: Å) between S_0 and S_1 geometries. (b) Individual bond order difference between S0 state and HOMO-LUMO excitation state. (c) The plot of HOMO. (d) The plot of LUMO.

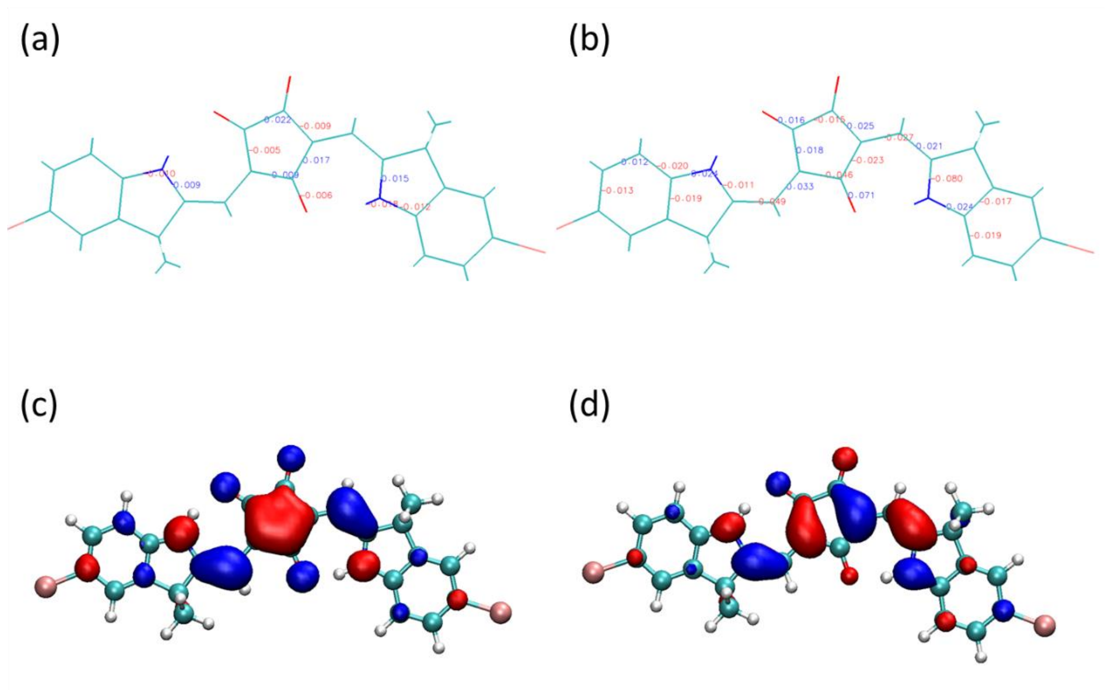

Figure S19. Results of high-level calculation for QALLOH. (a) Individual bond length difference (unit: Å) between S_0 and S_1 geometries. (b) Individual bond order difference between S0 state and HOMO-LUMO excitation state. (c) The plot of HOMO. (d) The plot of LUMO.

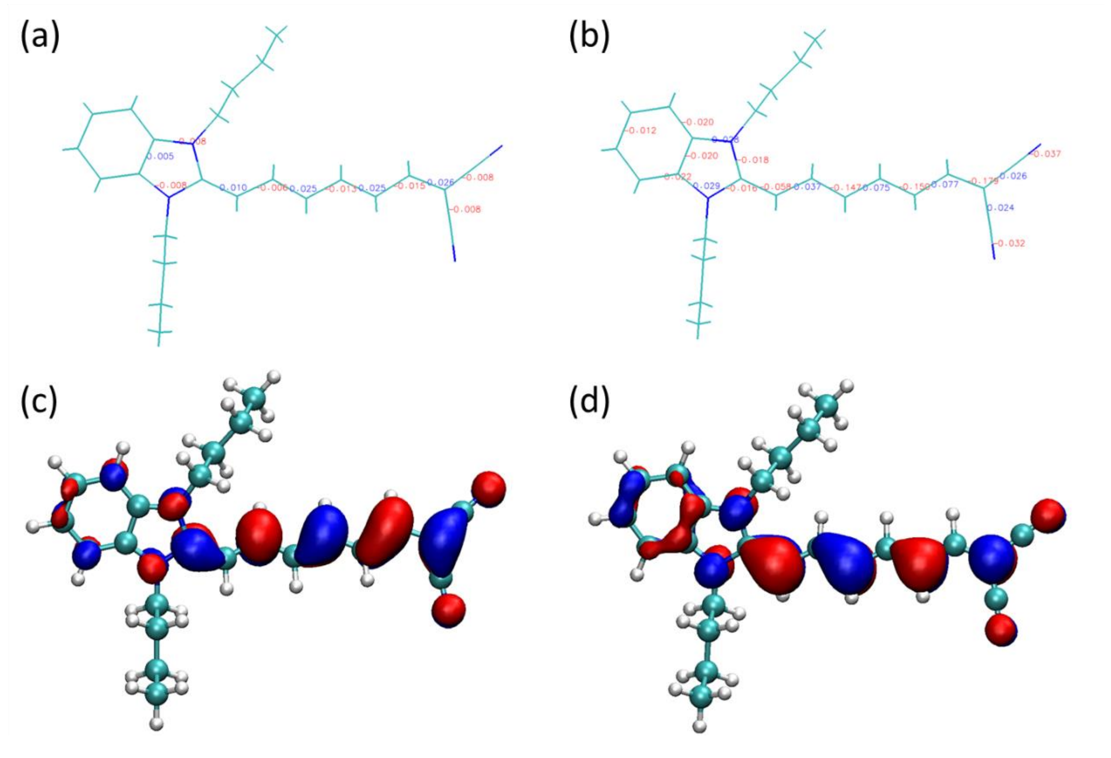

Figure S20. Results of high-level calculation for CITCUG. (a) Individual bond length difference (unit: Å) between S_0 and S_1 geometries. (b) Individual bond order difference between S0 state and HOMO-LUMO excitation state. (c) The plot of HOMO. (d) The plot of LUMO.

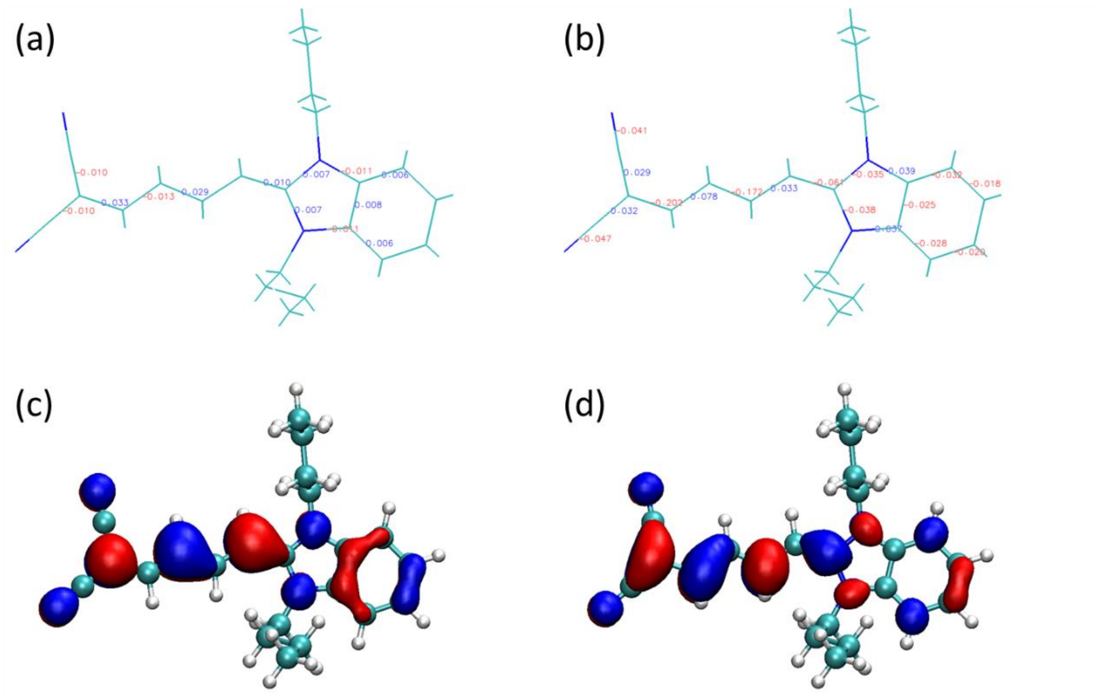

Figure S21. Results of high-level calculation for CITCOA. (a) Individual bond length difference (unit: Å) between S_0 and S_1 geometries. (b) Individual bond order difference between S0 state and HOMO-LUMO excitation state. (c) The plot of HOMO. (d) The plot of LUMO.

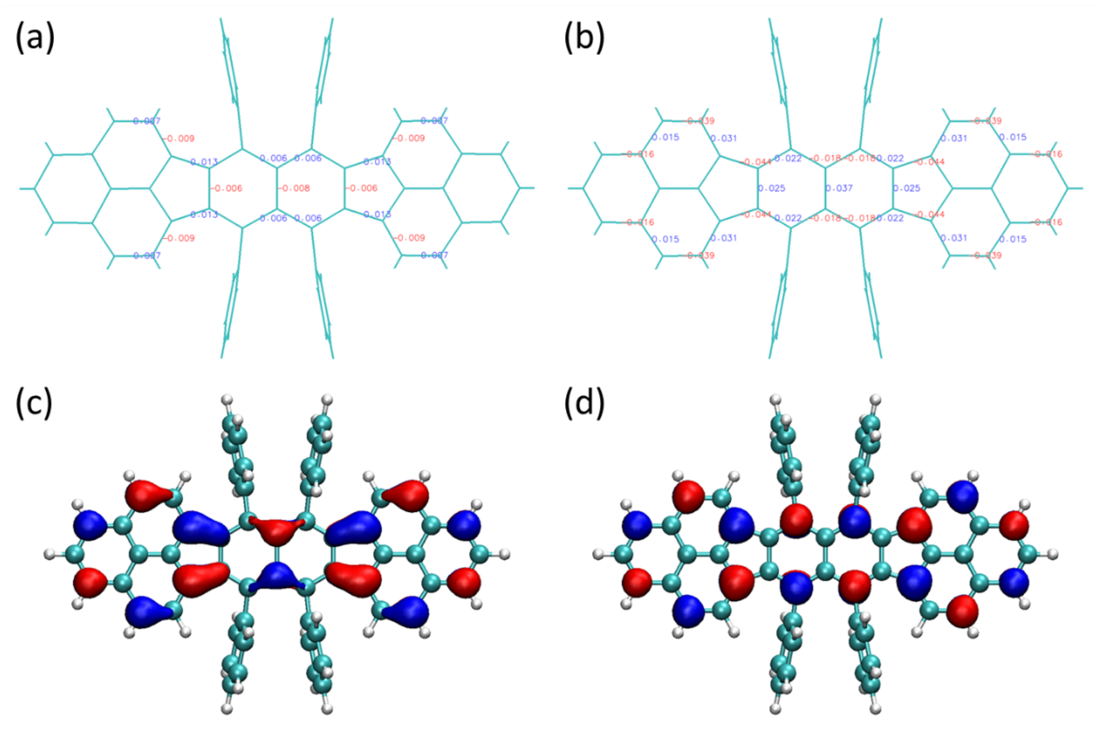

Figure S22. Results of high-level calculation for OPOPOZ. (a) Individual bond length difference (unit: Å) between S_0 and S_1 geometries. (b) Individual bond order difference between S0 state and HOMO-LUMO excitation state. (c) The plot of HOMO. (d) The plot of LUMO.

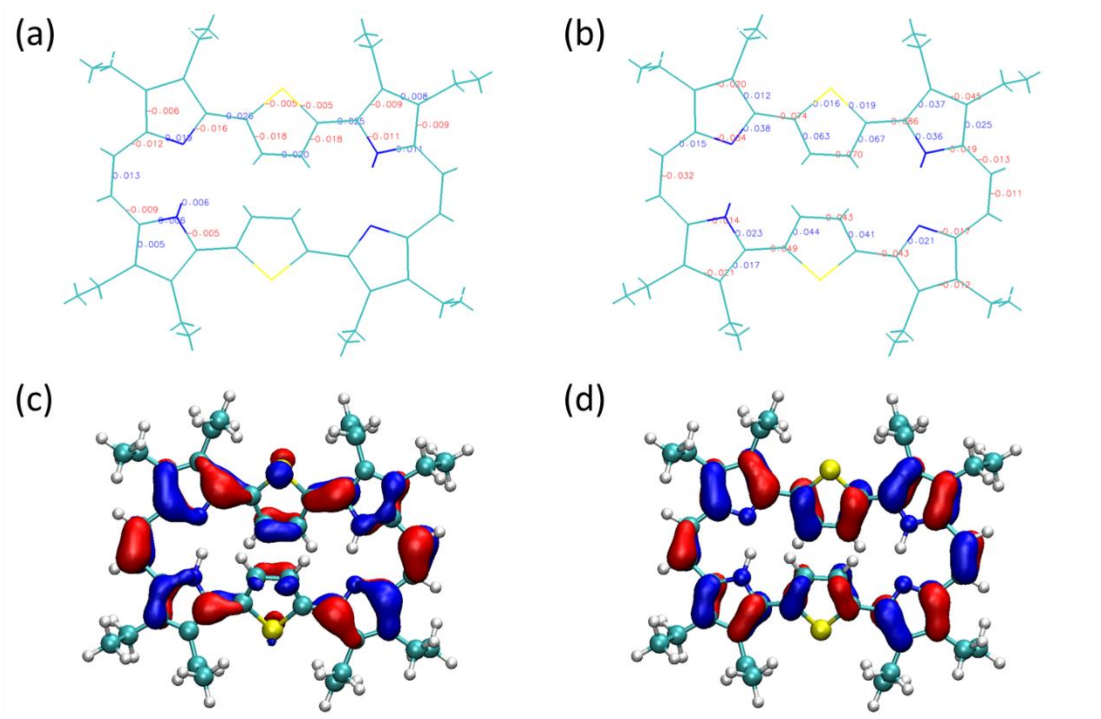

Figure S23. Results of high-level calculation for QIQSER. (a) Individual bond length difference (unit: Å) between S_0 and S_1 geometries. (b) Individual bond order difference between S0 state and HOMO-LUMO excitation state. (c) The plot of HOMO. (d) The plot of LUMO.

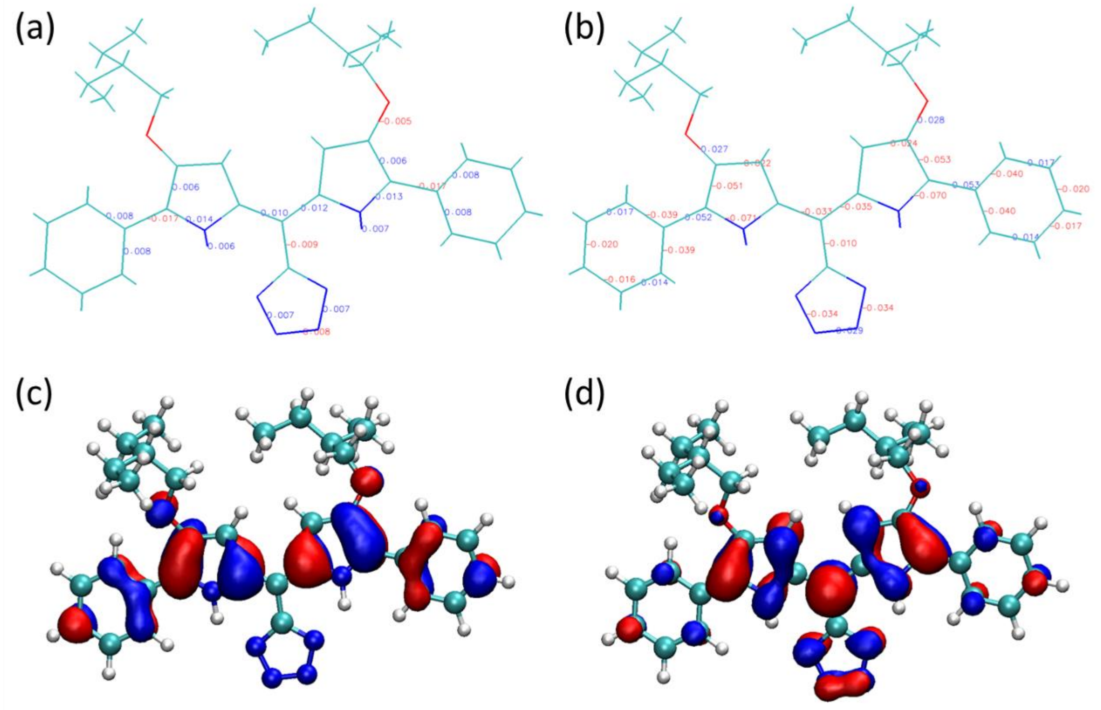

Figure S24. Results of high-level calculation for WOJWIE. (a) Individual bond length difference (unit: Å) between S_0 and S_1 geometries. (b) Individual bond order difference between S0 state and HOMO-LUMO excitation state. (c) The plot of HOMO. (d) The plot of LUMO.

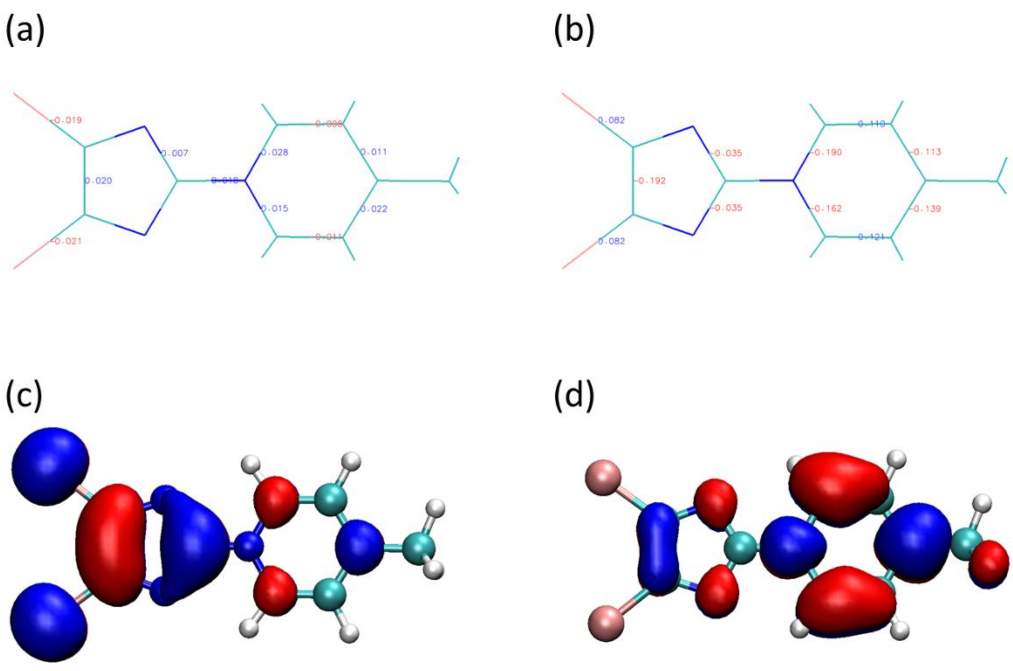

Figure S25. Results of high-level calculation for MUKKAE. (a) Individual bond length difference (unit: Å) between S_0 and S_1 geometries. (b) Individual bond order difference between S0 state and HOMO-LUMO excitation state. (c) The plot of HOMO. (d) The plot of LUMO.

In **Figure S25**, the positive bond order changes on the pyridine ring (0.110/0.121), which are maximal values among representative molecules, correspond to relatively small bond length changes (-0.008/-0.011), resulting in the small reorganization energy of MUKKAE molecules. And it may result from the Coulomb repulsion of the negative pyridine ring at the S_1 state (resulting from the CT character of the S_1 state).

Reference

(1) Xie, X.; Troisi, A. *Supporting data for this work*. 2023. https://github.com/XiaoyuUoL/ForceApproach (accessed 2023 13, March).
(2) Mayer, I. Charge, bond order and valence in the AB initio SCF theory. *Chem. Phys. Lett.* **1983**, *97* (3), 270-274.
(3) Mayer, I. Bond orders and valences from ab initio wave functions. *Int. J. Quantum Chem.* **1986**, *29* (3), 477-483. DOI: 10.1002/qua.560290320.
(4) Mayer, I. On bond orders and valences in the Ab initio quantum chemical theory. *Int. J. Quantum Chem.* **1986**, *29* (1), 73-84. DOI: 10.1002/qua.560290108.
