# Supplementary material for: Identification via Virtual Screening of Emissive Molecules with a Small Exciton–Vibration Coupling for High Color Purity and Potential Large Exciton Delocalization
Source: J Phys Chem Lett. 2023 Apr 27;14(17):4119–26. doi: 10.1021/acs.jpclett.3c00749 (PMC10165648; doi:10.1021/acs.jpclett.3c00749)
Supplement: Supplementary file 2 — jz3c00749_si_002.pdf [file jz3c00749_si_002.pdf]

jz-2023-00749s.R1

Name: Peer Review Information for "Identification via Virtual Screening of Emissive Molecules with Small Exciton-vibration Coupling for High Colour Purity and Potential Large Exciton Delocalisation"

First Round of Reviewer Comments

Reviewer: 1

Comments to the Author

In this work Xie and Troisi calculated the exciton relaxation energy for a large set of molecules and correlated its strength with the with the bonding/antibonding character of the orbitals that are most involved in the excitation (i.e. HOMO and LUMO for > 70% of the investigated molecules). Through a well-oiled virtual screening procedure that the authors employed in previous works, they identified and classified molecules with a small exciton reorganization energy (which are interesting for applications).

Though I think this work is very valuable to the community, I am a little concerned about the novelty of its conclusion and I would like to ask the authors to review their paper according to the following points before it can be published.

1) I feel the fact that the reorganization energy can be somehow attributed to the bonding/antibonding pattern of HOMO/LUMO orbitals was already around in the literature for some time (e.g., works from Shuai et al.). Recently, the same idea was discussed in J. Phys. Chem. A 124, 7644–7657 (2020), Chem. Sci. 13, 7181–7189 (2022), more qualitatively in Nat. Commun. 13, 2755 (2022) and in some other works. I think the authors should put their work better into perspective wrt what has been done already. They should clearly mention other people’s work and revise a little bit their claim of novelty (e.g., through the text and in the conclusion) in light of previous work.

2) The authors might also want to briefly discuss the role played by symmetry, if any, in determining the orbital shape, i.e., along the similar lines of what was done here: Chem. Sci. 13, 7181–7189 (2022).

3) This is a very minor comment, but I wonder if it would not be better to refer as exciton relaxation energy to the definition the authors give in equation 1 and 3. As the authors point out on page 5 the reorganization energy in Marcus theory would be twice the value they give in this work. I feel it’s slightly misleading calling equation 1 and 3 reorganization energy. Moreover, in the title they refer to exciton-vibration coupling, which is related to reorganization energy. I think might be a bit confusing for a reader the fact that the authors use a different terminology in the same context.

4) As the authors know, low reorganization energy does not necessarily imply large exciton delocalization (like the authors mention in their title). I wonder whether to make the paper more appealing – and to justify their title a bit better – the authors could not try to calculate excitonic couplings e.g. like they did in Chem. Mater. 33, 3368–3378 (2021), at least for the crystals with small molecular relaxation energies energy and to set up a TB Hamiltonian like they have done in that paper. The diagonal disorder could be

included from linear response theory using the relaxation energy calculated in this work (or calculating the local electron-phonon coupling constant, like they have done in other works). This way the author could find some sort of exciton size and clearly justify that they did indeed “identified emissive molecules with small exciton-vibration coupling … and large exciton delocalization”, like they report in the title. Otherwise, the authors might want to consider revisiting their title and choose one that reflects a bit better the message of the paper.

Author's Response to Peer Review Comments:

Dear Prof. Editor,

Please check the attachment of the response letter. Two manuscript files are also submitted, one of which ('manuscript-revised-color.docx') contains information about what we have changed (highlighted in red).

Best wishes!

Xiaoyu

Author's response for the manuscript ID jz-2023-00749s, *"Identification via Virtual Screening of Emissive Molecules with Small Exciton-vibration Coupling for High Colour Purity and Large Exciton Delocalisation"* by Xiaoyu Xie and Alessandro Troisi

We are grateful to the reviewer for reading the manuscript and providing constructive suggestions for its improvement. We report our response below with indications of the changes made in the manuscript and their position. For convenience, we submit a version of the manuscript with changes highlighted in red and address these changes in this response letter using *italics*.

Reviewer: 1

Recommendation: This paper is probably publishable, but major revision is needed; I do not need to see future revisions.

Comments:

In this work Xie and Troisi calculated the exciton relaxation energy for a large set of molecules and correlated its strength with the with the bonding/antibonding character of the orbitals that are most involved in the excitation (i.e. HOMO and LUMO for > 70% of the investigated molecules). Through a well-oiled virtual screening procedure that the authors employed in previous works, they identified and classified molecules with a small exciton reorganization energy (which are interesting for applications).

Though I think this work is very valuable to the community, I am a little concerned about the novelty of its conclusion and I would like to ask the authors to review their paper according to the following points before it can be published.

1) I feel the fact that the reorganization energy can be somehow attributed to the bonding/antibonding pattern of HOMO/LUMO orbitals was already around in the literature for some time (e.g., works from Shuai et al.). Recently, the same idea was discussed in J. Phys. Chem. A 124, 7644–7657 (2020), Chem. Sci. 13, 7181–7189 (2022), more qualitatively in Nat. Commun. 13, 2755 (2022) and in some other works. I think the authors should put their work better into perspective wrt what has been done already. They should clearly mention other people's work and revise a little bit their claim of novelty (e.g., through the text and in the conclusion) in light of previous work.

We fundamentally agree with this comment. We had no intention to claim originality on this aspect of the paper, which is also covered in some undergraduate textbooks. We revise the discussion on page 5, including the statement "This is not a novel idea" and a brief discussion of other recent works including those proposed by the referee. However, we also stress that what is new here is the *quantitative* assessment of how far one can go using a measure of bonding character change in conjunction with a large data set. We stress in the revised manuscript that quantification of correlation is important because there are other "old" proposals, such as the correlation between delocalisation and reorganization energy, that turn out to be not very useful.

"However, there is only a very weak correlation between measures of delocalization of frontier orbitals (e.g., inverse participation ratio) and reorganization energy (correlation coefficient $R = 0.19$), as reported in SI, highlighting the importance of using large data set to determine structure-property relations that are truly useful in practive."

"The summation extends over all pairs of chemically connected atoms. This idea, related to the bonding/antibonding pattern of HOMO/LUMO, is not novel and has already been covered in the literature. [11,33-35] For example, the bond-order–bond-length (BOBL) relationship was used to elucidate reorganization energy in recent work.[35] However, the predictive ability of this descriptor based on a large dataset has not been presented before and, as we have seen, this is critical to establish its usefulness."

2) The authors might also want to briefly discuss the role played by symmetry, if any, in determining the orbital shape, i.e., along the similar lines of what was done here: Chem. Sci. 13, 7181–7189 (2022).

Following this comment, we have considered whether we can add some insight on this point based on our dataset. We did not notice any correlation between the presence of symmetry elements (in addition to the plane in planar molecules) and reorganization energy. Clearly, this is not in contradiction with the observation of the Chem Sci paper but a reflection of the "unbiased" dataset used with limited number of symmetric molecules. We added a comment on pages 8-9 of the manuscript.

"Finally, we should also note that some important effects are best captured with a set of molecules designed ad hoc, which are difficult to analyse based on the large dataset in our case. For example, Pi-Tai Chou et al.[33] found that molecular reflection symmetry plays an important role in reducing reorganization energy in linear cyanine systems, while we have very few symmetric molecules."

3) This is a very minor comment, but I wonder if it would not be better to refer as exciton relaxation energy to the definition the authors give in equation 1 and 3. As the authors point out on page 5 the reorganization energy in Marcus theory would be twice the value they give in this work. I feel it's slightly misleading calling equation 1 and 3 reorganization energy. Moreover, in the title they refer to exciton-vibration coupling, which is related to reorganization energy. I think might be a bit confusing for a reader the fact that the authors use a different terminology in the same context.

The double nomenclature is a bit unfortunate but useful because reorganization energy is more common in exciton transport and exciton-vibration coupling is more common in the study of emissive materials (we target both applications – having a distinct set of readers). To ease the reading, we clarify that the two concepts are strongly related (i) in the revised abstract where both names are now used and (ii) the first time the equations are introduced (page 2).

(i) *"A sequence of quantum chemical computations of increasing accuracy was used in this work to identify molecules with small exciton reorganization energy (exciton-vibration coupling), ..."*

(ii) *"The most common way to evaluate reorganization energy (a measure of exciton-vibration coupling) is by using its definition, …"*

4) As the authors know, low reorganization energy does not necessarily imply large exciton delocalization (like the authors mention in their title). I wonder whether to make the paper more appealing – and to justify their title a bit better – the authors could not try to calculate excitonic couplings e.g. like they did in Chem. Mater. 33, 3368–3378 (2021), at least for the crystals with small molecular relaxation energies energy and to set up a TB Hamiltonian like they have done in that paper. The diagonal disorder could be included from linear response theory using the relaxation energy calculated in this work (or calculating the local electron-phonon coupling constant, like they have done in other works). This way the author could find some sort of exciton size and clearly justify that they did indeed "identified emissive molecules with small exciton-vibration coupling … and large exciton delocalization", like they report in the title. Otherwise, the authors might want to consider revisiting their title and choose one that reflects a bit better the message of the paper.

To address this valid point, we propose a change of title and additional clarification. In this work, we address molecular properties rather than solid state properties and so "colour purity" is something addressed directly while "large exciton delocalisation" is something also depending on the excitonic coupling (we did not compute it here but we ensured it is large because the oscillator strength is large) and the non-local exciton-phonon coupling (which currently cannot be addressed in a high-throughput fashion). This clarification is introduced on page 4 (also shown below), and the title is changed to "Identification via virtual screening of emissive molecules with small exciton-vibration coupling for high colour purity and potential large exciton delocalisation".

"…considering only molecules with S_1 oscillator strength larger than 0.5 (since a bright emissive state is required by all the applications mentioned in the introduction and the finding can be tested more easily). It should be noted that this work only directly addresses molecular rather than solid state properties, as it was noted that the extent of exciton delocalisation is influenced not only by the excitonic coupling but also the non-local exciton-phonon coupling.[25,26]"
